# MicroRNA-mediated regulation of reactive astrocytes in central nervous system diseases

**DOI:** 10.3389/fnmol.2022.1061343

**Published:** 2023-01-12

**Authors:** Yuansheng Fan, Hui Huang, Junfei Shao, Weiyi Huang

**Affiliations:** Department of Neurosurgery, The Affiliated Wuxi People’s Hospital of Nanjing Medical University, Wuxi, Jiangsu, China

**Keywords:** central nervous system, microRNAs, ischemic stroke, spinal cord injury, neurodegenerative disease, reactive astrocytes

## Abstract

Astrocytes (AST) are abundant glial cells in the human brain, accounting for approximately 20–50% percent of mammalian central nervous system (CNS) cells. They display essential functions necessary to sustain the physiological processes of the CNS, including maintaining neuronal structure, forming the blood–brain barrier, coordinating neuronal metabolism, maintaining the extracellular environment, regulating cerebral blood flow, stabilizing intercellular communication, participating in neurotransmitter synthesis, and defending against oxidative stress et al. During the pathological development of brain tumors, stroke, spinal cord injury (SCI), neurodegenerative diseases, and other neurological disorders, astrocytes undergo a series of highly heterogeneous changes, which are called reactive astrocytes, and mediate the corresponding pathophysiological process. However, the pathophysiological mechanisms of reactive astrocytes and their therapeutic relevance remain unclear. The microRNAs (miRNAs) are essential for cell differentiation, proliferation, and survival, which play a crucial role in the pathophysiological development of CNS diseases. In this review, we summarize the regulatory mechanism of miRNAs on reactive astrocytes in CNS diseases, which might provide a theoretical basis for the diagnosis and treatment of CNS diseases.

## 1. Introduction

Astrocytes are one of the most significant cellular components and represent the largest number of cells in the CNS, accounting for 20–50% of all CNS cells, which play an important role in maintaining endocytosis of extracellular fluid, ions, and transmitters (Verkhratsky and Nedergaard, 2018), feeding neurons (Magistretti and Allaman, 2018), regulating local blood flow (Mac Vicar and Newman, 2015), helping regulate interstitial fluid drainage (Plog and Nedergaard, 2018) as well as synaptic development and plasticity (Allen and Eroglu, 2017). There are two types of astrocytes, fibrous and protoplasmic astrocytes, based on the content of glial filaments and the shape of cytoplasmic protrusions. Fibrous astrocytes are primarily distributed in the cerebral cortex, with elongated protrusions and fewer branches. Protoplasmic astrocytes are mainly in gray matter, with thick and short protrusions and many branches. However, this traditional classification ignores the heterogeneity of astrocytes, which exhibit transcriptomic and functional heterogeneity in different brain regions and cortical layers (Yeh et al., 2009; Zeisel et al., 2015; Lanjakornsiripan et al., 2018).

Reactive astrocytes, induced by brain injuries and disease, undergo a series of molecular, morphological, and functional alterations such as inflammatory factor release (Hasel et al., 2021), glial scar formation (Sofroniew and Vinters, 2010), downregulation of glutamate transporter-1 (GLT-1) expression (Tanaka et al., 1997), and upregulation of water channel protein aquaporin 4 (AQP4; Kitchen et al., 2020). These reactive astrocytes are crucial in regulating the neuron, microglia, and CNS microenvironment after SCI, stroke, neurodegeneration, and others (Liddelow and Barres, 2017). Studies have found that miRNAs play a profound role in mediating the activities and functions of reactive astrocytes in recent years. miRNAs are a class of endogenous small non-coding RNA molecules (20–25 nucleotides). Mature miRNAs under the action of RISC complex, namely RNA-induced silencing complex, work with the 3’UTR specific base of target gene mRNA sequence to cause the degradation of target gene mRNA or to inhibit the translation of target gene mRNA, thereby regulating the post-transcriptional expression of genes. In addition, miRNAs may interact with the target gene mRNA 5′-UTR coding sequences and the target promoter region. Researchers initially considered miRNAs critical players in CNS development (Worringer et al., 2014). Nonetheless, as research progress, studies have identified miRNAs as essential in CNS diseases. miRNAs in these diseases are often altered by genomic events such as gene mutations, deletion amplification, or transcriptional changes, or by mutations or downregulation of enzymes that regulate miRNA biogenesis, resulting in biogenetic defects (Bartel, 2004; Lin and Gregory, 2015; Rupaimoole et al., 2016). Studies have been on miRNAs as potential targets to regulate glioma cells’ proliferation, migration, and invasion. Meanwhile, mounting data suggests that miRNAs could exert a range of protective effects by regulating the activities and functions of reactive astrocytes in CNS diseases such as stroke, SCI, and neurodegeneration (Hong et al., 2014; Duan et al., 2021; Li L. et al., 2021).

This article reviews the research on miRNAs in regulating the activities and functions of reactive astrocytes and summarizes the relevant mechanisms of miRNAs regulating reactive astrocytes in CNS diseases to provide a theoretical basis for diagnosing and treating CNS diseases.

## 2. MicroRNAs regulate the activities and functions of reactive astrocytes

### 2.1. Apoptosis and autophagy

Apoptosis is the orderly process of cell death through intracellular genetic mechanisms that eventually lead to the degradation and digestion of all cellular components by other living cells. Autophagy, a catabolic process, could degrade and recycle dysfunctional organelles and proteins (Fricker et al., 2018). During brain ischemia, astrocytes undergo autophagy, which represents the cell’s attempt to cope with stress and protects the cell from apoptosis (Mo et al., 2020). Thus, autophagy might protect astrocytes from apoptosis injured by ischemia. In a study, circ_0025984 expression is significantly reduced in astrocytes during cerebral ischemia, and its overexpression strongly inhibits ischemia-induced astrocyte apoptosis to suppress brain injury. And it has been shown that the protective effect of circ_0025984 is associated with the inhibition of apoptosis by directly targeting the miR-134-3p/TET1/ORP150 pathway (Zhou et al., 2021). Similarly, miR-30d, which targets BECLIN1, is essential in the interplay between astrocyte autophagy and apoptosis following oxygen–glucose deprivation reperfusion (OGD/R). miR-30d could be seen as a novel target to attenuate cellular damage under hypoxic–ischemic conditions (Zhao et al., 2017).

### 2.2. Pathophysiological functions

**Figure 1:**
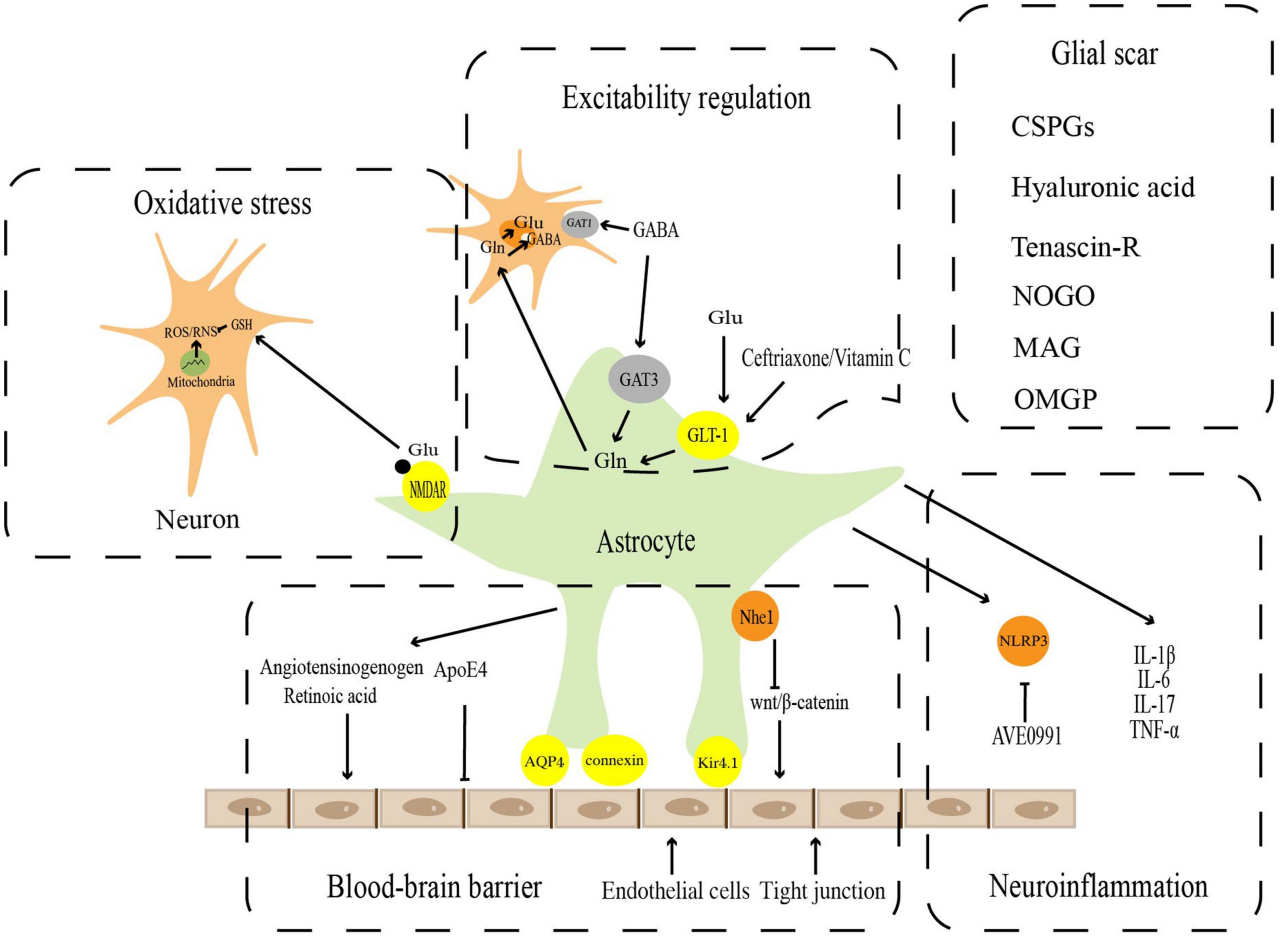
Pathophysiological functions of reactive astrocytes. Astrocytes play a key role in the maintenance of CNS homeostasis, including blood–brain barrier, excitatory regulation, oxidative stress, neuroinflammation, and Glia scar.

Astrocytes have long been identified essential mediators in maintaining the CNS microenvironment’s homeostasis under physiological conditions and performing critical physiological functions. However, studies over the past few decades have revealed that astrocytes undergo significant cellular, molecular, and functional alterations to transform into reactive astrocytes following pathological injury in the CNS. These changes are used to regulate the blood–brain barrier, excitability regulation, oxidative stress, neuroinflammation, and glial scar in response to the homeostatic dysregulation (Figure 1).

#### 2.2.1. Blood–brain barrier

Vascular endothelial cells, astrocytes, and peripheral cells are participated in the maturation and maintenance of the blood–brain barrier, which is critical for the homeostasis and function of the CNS (Weiss et al., 2009; Daneman and Engelhardt, 2017; Liebner et al., 2018). The blood–brain barrier, which covers approximately 99% of the cerebrovascular surface, regulates blood–brain barrier integrity and cerebral blood flow (Iadecola and Nedergaard, 2007; Masamoto et al., 2015), and forms a secondary barrier that further prevents peripheral immune cells from non-neural tissue from entering the CNS (Sofroniew and Vinters, 2010). Gap junction proteins, such as connexin, AQP4, and Kir4.1 channels, which are specific dynamic sites regulating ion and water flow, and are essential for blood–brain barrier function, are abundant in the astrocyte end-food (Alvarez et al., 2013; Boulay et al., 2016). The perivascular astrocytes polarize in SCI (Nesic et al., 2006), ischemic stroke (Manley et al., 2000), and tumors (Verkman et al., 2008) to upregulate the expression of AQP4 on cell membranes, resulting in end-foot edema and vascular regression. This causes gap junctions to open and the blood–brain barrier to be damaged, which induces a series of secondary brain injuries (Xiang et al., 2016; Haley and Lawrence, 2017). Apolipoprotein E (ApoE) is a multifaceted secreted molecule synthesized in the CNS by astrocytes and microglia (Lanfranco et al., 2021). ApoE has been shown to impact the integrity of the blood–brain barrier (Montagne et al., 2020). Specific expression of ApoE4 alleles but not APOE2 or APOE3 derived from astrocytes results in disrupting blood–brain barrier integrity, increasing matrix metallopeptidase 9 (MMP9), impairing tight junctions, and reducing vascular coverage (Jackson et al., 2022). Wnt growth factor secreted by astrocytes maintains the integrity of the blood–brain barrier by promoting Wnt/β-linked protein activity in endothelial cells (Guérit et al., 2021). It has recently been shown that astrocytes could repair the blood–brain barrier with Nhe1 protein deficiency *via* the Wnt/β-catenin signaling pathway (Song et al., 2021). In addition, the angiotensinogen released by astrocytes could be converted into angiotensin to regulate the expression of occludin, one of the major tight junction proteins, *in vivo* (Wosik et al., 2007). Meanwhile, retinoic acid secreted by astrocytes and radial glial cells could also increase the levels of tight junction components such as ZO-1 (Mizee et al., 2013).

Ischemic stroke is caused by reduced or blocked blood flow to the CNS due to a distant embolus, *in situ* thrombus, or atherosclerotic plaque formation blocking an artery in the brain. miRNAs and astrocytes are key players in the progression of ischemic stroke. Their regulatory roles in ischemia-induced oxidative stress (Li L. et al., 2021), and apoptosis-related gene expression (Zhao et al., 2017) have been demonstrated. Growing evidence suggests that AQP4 plays a vital role in ischemic brain injury (Zhang et al., 2015). AQP4 exacerbates cerebral ischemia by increasing brain edema (Zeng et al., 2010; Thrane et al., 2011; Fukuda and Badaut, 2012). The study has found that miR-130b promotes neuroprotection by binding to the 3’UTR region of AQP4 mRNA and downregulating AQP4 levels in astrocytes at the post-transcriptional stage miR-130b may be a novel target for treating ischemic stroke (Zheng et al., 2017). Another miRNA that also targets AQP4 is miR-145. miR-145 is essential in protecting astrocytes from ischemic injury by downregulating AQP4 expression.

#### 2.2.2. Excitability regulation

Astrocytes are involved in buffering some neurotransmitters released by neurons, such as glutamate and gamma-aminobutyric acid (GABA), to eliminate their continued effects on neurons and provide precursors for amino acid neurotransmitters (Sofroniew and Vinters, 2010). Glutamate is the main excitatory transmitter in the brain and spinal cord. The excessive accumulation of glutamate in the synaptic gap leads to excitatory damage. There are five mammalian excitatory amino acid transporters, EAAT1, EAAT2, EAAT3, EAAT4, and EAAT5 (Arriza et al., 1994, 1997; Fairman et al., 1995). The first 2 isoforms, EAAT1 and EAAT2, are known as GLAST (Storck et al., 1992)and GLT-1 (Pines et al., 1992), respectively, which play critical roles in regulating glutamate homeostasis. Under physiological conditions, astrocytes take up glutamate from the synaptic gap *via* glutamate transporters and convert it into glutamine to prevent excitatory damage from the overaccumulation of glutamate in the synaptic gap. Glutamine could also be a substrate for glutamate resynthesis by neurons (Hamilton and Attwell, 2010). Following brain injury, the glutamate system, which relies on astrocytes, changes in several ways, including epigenetic regulation of the GLT-1 and GLAST promoters, abnormal histone methylation leading to gene dysfunction (Chisholm et al., 2015), and S-nitrosylation of GLT-1 leading to reduced activity (Yamada et al., 2006). As brain damage progresses, ATP levels in astrocytes drop, inducing glutamate transporter reversal and further exacerbating glutamate excitotoxicity (Zeevalk et al., 1998). *In vitro* and *in vivo* (Weller et al., 2008) experiments have demonstrated that upregulation of GLT-1 in astrocytes by ceftriaxone (Ouyang et al., 2007) or adenoviral vectors (Harvey et al., 2011) could reduce the area of cerebral infarction, attenuate neurological impairment, and offer neuroprotective effects against ischemic stroke. Ceftriaxone exerts the similar influence in epilepsy disease, which could upregulate GLT-1 expression by reducing glutamate in the hippocampus, mitigating the onset of seizures and the impairment of learning and memory in the chronic phase (Ramandi et al., 2021). Vitamin C acts as a neuroprotective agent by promoting astrocyte GLT-1 expression, reducing glutamate aggregation, and attenuating excitatory damage in Parkinson’s disease (PD)(Zeng et al., 2022).

GABA is the primary inhibitory neurotransmitter in the brain, which is mainly transported from the synaptic gap by specific γ-aminobutyric acid transporters (GATs) expressed by neurons and surrounding astrocytes. Presynaptic neurons mainly express GAT subtype 1 (GAT1), and astrocytes mainly express GAT subtype 3 (GAT3; Zhou and Danbolt, 2013). Previous studies have found that presynaptic nerve endings express high concentrations of GAT1 (Conti et al., 2004). GABA is primarily transported from the synaptic gap by GAT1 on presynaptic neurons and stored in presynaptic neurons for later release (Schousboe et al., 2013). These imply that neurons play an imperative role in maintaining synaptic GABA homeostasis. However, recent studies have demonstrated that in contrast to neuronal GAT1, selectively inhibition of astrocyte GAT3 reduces GABA uptake of astrocyte, significantly decreases GABA metabolism, and inhibits astrocyte glutamine production (Andersen et al., 2020). The results imply that astrocytes are involved in GABA metabolism and serve a crucial metabolic function in maintaining GABA homeostasis. In flinders sensitive line (FSL) rat models, reactive astrocytes promote GABA synthesis and release, which results in increased tonic GABA inhibition. The increased synthesis and release of GABA could be blocked by monoamine oxidase B (MAO-B), which reduces tonic inhibition and has antidepressant effects (Srivastava et al., 2020). Few studies have reported that miRNAs could regulate GABA metabolism to exert neuroprotective effects in neurological disorders, which could be a novel horizon for the future.

In cerebral ischemia-induced excitotoxicity and neuronal damage, the upregulation of astrocyte GLT-1 is a potential therapeutic target. MiR-107 and GLT-1 expression were discovered to be correlated in a rat model of focal cerebral ischemia/reperfusion (I/R) damage. miR-107 inhibits GLT-1 expression, which also causes extracellular glutamate to build up (Yang et al., 2014). Additional research has demonstrated that magnesium lithospermate B (MLB) could protect the rat brain from excitatory neurotoxicity by regulating miR-107/GLT-1 and reducing extracellular glutamate aggregation after I/R (Yang et al., 2015). Similar report of miR-124 downregulation in a cerebral ischemia model has been made. GLT-1 expression of astrocytes is dramatically elevated with OGD/R when miR-124 expression improves (Huang et al., 2019).

PD is an age-related movement disorder characterized pathologically by progressive dopaminergic cell death (Olanow and Tatton, 1999). Different mechanisms of dopaminergic neuron death in PD include genetic factors (Miklya et al., 2014), environmental factors (Aguirre-Gamboa et al., 2016; Ter Horst et al., 2016), neuroinflammation (Hirsch and Hunot, 2009), and glutamate excitotoxicity (Ambrosi et al., 2014; Gardoni and Di Luca, 2015). Because the etiology of sporadic PD is unknown, there is a lack of effective therapeutic measures. Among the several etiological theories of PD, glutamate excitotoxicity theory has recently taken center stage (Chang et al., 2020). Glutamate transporters play a significant role in removing excess glutamate from the synaptic gap. GLT-1 is the critical factor in the development of PD, as it is responsible for the uptake of nearly 90% of synaptic glutamate (Storck et al., 1992; Zhang et al., 2017). Therefore, regulating GLT-1 expression in astrocytes during PD by microRNAs to delay the progression or exacerbation of PD is a potential research direction. It has been established that miR-543-3p directly regulated GLT-1 mRNA (SLC1A2 gene), and the inhibition of miR-543-3p upregulated GLT-1 protein expression and function, alleviating dyskinesia in PD models (Wu et al., 2019). The miR-30 family, which includes the mature miRNA sequences miR-30a, miR-30b, miR-30c-1, miR-30c-2, miR-30d, and miR-30, is made up of six distinct miRNAs (Chang et al., 2008). A study has reported that miR-30a-5p, which was upregulated in MPTP-treated mice (a mouse model of PD), decreased GLT-1 expression and function through the ubiquitin-proteasome degradation pathway, thereby participating in the pathological process of PD. This study provides strong evidence for miR-30a-5p as a potential therapeutic target for PD (Meng et al., 2021).

Amyotrophic lateral sclerosis (ALS) is a highly progressive disease characterized by losing motor neurons in the brain and spinal cord. The occurrence of ALS is driven spontaneously by motor neuron damage, and astrocytes strongly influence the rate of disease progression (Al-Chalabi and Hardiman, 2013). miR-218 is abundantly enriched in motor neurons and released extracellularly into the cerebrospinal fluid in ALS rats. Motor neuron-carried miR-218 could be taken up by astrocytes and downregulate EAAT2 in astrocytes. In ALS mice, the inhibition of miR-218 with antisense oligonucleotides attenuates the loss of EAAT2 and other miR-218-mediated changes, providing meaningful evidence for microRNA-mediated communication between neurons and astrocytes *in vivo* (Hoye et al., 2018). These findings imply that microRNAs from dead neurons could directly alter the glial phenotype and lead to astrocyte dysfunction, exacerbating additional neuronal damage.

#### 2.2.3. Oxidative stress

During neurotransmission, reactive oxygen and nitrogen are inevitably created. Nerve cells generate endogenous reactive oxygen species (ROS) *via* the mitochondrial electron transport chain and the NADPH oxidation pathway, which are subsequently catalyzed by nitric oxide synthase (NOS) to produce reactive nitrogen species (RNS) (Cobley et al., 2018). Ascorbic acid and glutathione (GSH) are the two main components of the antioxidant system in brain tissues (Makar et al., 1994). Glutamate binds to N-methyl-D-aspartate (NMDA) receptors (NMDAR) on astrocytes to promote ectopic nuclear factor erythroid 2-related factor 2 (NRF2) to the nucleus, inducing antioxidant gene expression and GSH synthesis. This promotes intraneuronal GSH transport. The increasing GSH exerts a neuroprotective impact by improving the antioxidant capacity of neurons (Jimenez-Blasco et al., 2015; Skowrońska et al., 2019). In reactive astrocytes, the expression of NMDAR is usually upregulated. Two astrocyte NMDAR subunits, GluN2A and GluN2B, showed elevated expression in the transient ischemia hippocampus from day 3 and reached a peak at day 28 (Krebs et al., 2003). This upregulation of NMDAR might represent a neuroprotective response in reactive astrocytes (Jimenez-Blasco et al., 2015). The NMDAR subunit GluN3A has been found to increase in astrocytes in the middle cerebral artery occlusion (MCAO) mouse model (Dzamba et al., 2015), which could inhibit NMDARs activity and suppress the overall antioxidant capacity of astrocytes. However, there are few studies on regulating NMDAR on astrocytes by microRNAs to achieve neuroprotective effects. This may be a worthy direction for future research. Another antioxidant, ascorbic acid, which similarly shields neurons from oxidative stress, is also stored in astrocytes. As GSH synthesis declines in neurons, astrocytes manufacture and release ascorbic acid, which is then carried into neurons (Allaman et al., 2011). Recent research has shown that DJ-1, an essential antioxidant factor mainly produced by reactive astrocytes, exerts neuroprotective functions during ischemic injury by upregulating NRF2 and GSH expressions (Peng et al., 2019).

Mitochondria are central to ischemic cell death, which could regulate oxidative stress, ATP production, and intracellular calcium handling (Verdejo et al., 2012). Mitochondrial dysfunction promotes I/R injury (Stary et al., 2016). The assembly of the cytochrome C oxidase complex, which regulates ATP synthesis and mitochondrial biogenesis, relies on cytochrome C oxidase IV (COX IV) (Li et al., 2006). By targeting COX4I1, a COX IV isoform that is enriched in the brain, upregulating miR-338 in cerebral ischemia hinders COX IV protein synthesis and mitochondrial complex IV activity resulting in mitochondrial dysfunction and decreasing the ability of astrocytes to handle oxidative stress, and ultimately exacerbating ischemic brain injury (Li L. et al., 2021).

#### 2.2.4. Neuroinflammation

While astrocytes typically sustain neuronal function and survival through multiple mechanisms, these homeostatic functions are frequently impaired in neurological disorders. Inflammatory factors released by reactive astrocytes could hasten neuronal damage (Van Damme et al., 2007; Cassina et al., 2008; Ferraiuolo et al., 2011; Madji Hounoum et al., 2017).

Alzheimer’s disease (AD) is the most common cause of dementia worldwide (Wolters and Ikram, 2018), accounting for 50–70% of all cases (Querfurth and LaFerla, 2010). Amyloid β (Aβ) deposition into Aβ plaques and the formation of neurogenic fiber tangles (NFTs) composed of hyperphosphorylated tau proteins are the main neuropathological features of AD (Polanco et al., 2018). Astrocytes play a major role in regulating neuroinflammation in AD (Kwon and Koh, 2020). Activation of the NLRP3 inflammasome in astrocytes is involved in the pathogenesis of AD by triggering an inflammatory response in the brain (Heneka et al., 2013). Chronic neuroinflammation might lead to the destruction of neurons and synapses, which subsequently contribute to the deterioration of cognitive function, even if a moderate inflammatory response is necessary for the brain to clear Aβ. Previous observations showed that anti-inflammatory therapy effectively halted disease progression in animal models of AD (Brod, 2022). The inhibition of NLRP3 inflammatory vesicles might be a novel intervention for treating AD. The Ang-(1–7) analog AVE0991 could block astrocyte NLRP3 inflammasome-mediated neuroinflammation through the SNHG14/miR-223-3p/NLRP3 pathway to exert neuroprotective effects in APP/PS1 mice. These findings reveal that miR-223-3p plays a key role in regulating astrocyte-mediated inflammation in AD. More significantly, the study identifies Ang-(1–7)-targeted SNHG14/miR-223-3p as an inhibitor of neuroinflammation in AD and reveals the therapeutic potential of its non-peptide analog AVE0991 (Duan et al., 2021).

In SCI, the primary injury results in the death of local neurons and glial cells within minutes to hours. A neuroinflammatory reaction-induced secondary damage follows immediately after that (Fleming et al., 2006). The occurrence and progression of SCI are significantly influenced by the inflammatory response. Following SCI, inflammatory cells (i.e., T cells, macrophages, and microglia) are recruited to the site of injury and clear cellular debris during the acute SCI phase (Ahuja et al., 2017). During the SCI, reactive astrocytes emit inflammatory factors, such as interleukin (IL)-17, IL-6, and tumor necrosis factor alpha (TNF-α), which might be in concert with various inflammatory mediators and other cytokines to aggravate the spinal cord damage further. A study has found that the expression of IL-1β, IL-6, and TNF-α in the serum of rats with SCI transfected with miR-136-5p was significantly higher than that of the control group, while the protein expression of A20 was significantly reduced and the expression of p-NF-κB was elevated. These results suggest that miR-136-5p could aggravate SCI injury by promoting the release of inflammatory factors from reactive astrocytes *via* the NF-κB/A20 signaling pathway. Silencing miR-136-5p could effectively diminish inflammatory factors and chemokines and protect the spinal cord through the NF-κB /A20 signaling pathway *in vivo* and *in vitro* (He et al., 2017). According to studies, miR-140 targets Brain-derived neurotrophic factor (BDNF) to regulate astrocyte proliferation through the PI3K/AKT pathway. miR-140 could inhibit the expression of BDNF, IL-6, and Transforming growth factor-alpha (TGF-α) in a lipopolysaccharide (LPS)-induced injury model. Therefore, miR-140/BDNF is expected to be a target for inhibiting the reactive proliferation of astrocytes and the release of inflammatory factors after SCI (Tu et al., 2017).

#### 2.2.5. Glial scar

Glial scar is widespread in the pathophysiological development of CNS diseases and consists mainly of proliferating and migrating reactive astrocytes, microglia, and oligodendrocyte precursor cells (Adams and Gallo, 2018). The formation of glial scar is accompanied by changes in the expression of several related molecules: (1) glycosaminoglycan (GAG) proteoglycans, one of the main components of chondroitin sulfate proteoglycans (CSPGs), are the most abundant components of glial scar and the main inhibitors of axonal regeneration (He et al., 2020); (2) other extracellular matrix components: high molecular weight hyaluronic acid and Tenascin-R regulate the formation of glial scar and inhibit axonal growth (Khaing et al., 2011); (3) myelin-associated glycoproteins, including Nogo, myelin-associated glycoprotein (MAG), and oligodendrocyte-myelin glycoprotein (OMgp; Ohtake and Li, 2015). CSPGs are composed of core protein and GAG chain attachment sites, including lectin, phosphoglycan, and NG2 (Dyck and Karimi-Abdolrezaee, 2015). As they do during development, CSPGs effectively inhibit regenerating axons (McKeon et al., 1991). Further studies have demonstrated that the GAG residues of CSPGs interact with various neuronal developmental inhibitory receptors such as Nogo receptors 1 and 3, protein tyrosine phosphatase, and leukocyte common antigen-associated phosphatase receptor, thereby inhibiting axon regeneration (Shen et al., 2009; Dickendesher et al., 2012). Chondroitinase ABC greatly lessens the inhibitory effect of CSPGs on axon growth by eliminating the GAG chain of CSPGs or by degrading the core proteins of CSPGs with matrix metalloproteinases (MMPs) after cerebral ischemia in rats (Bradbury and Carter, 2011; Cua et al., 2013; Mukherjee et al., 2020). In addition to its effect on axons, glial scar inhibits endogenous myelin reformation. Endothelin-1 (ET-1), a negative regulator of differentiation and functional myelin formation in NG2 glial cells, raises Jagged1 expression in reactive astrocytes, activates Notch signaling in neighboring NG2 glial cells, and hinders their differentiation (Hammond et al., 2014, 2015). After focal demyelination of the corpus callosum, it has been discovered that blocking ET-1 signaling by pharmacological or genetic pathways promotes the differentiation of NG2 glial cells into oligodendrocytes and supports endogenous myelin formation (Adams and Gallo, 2018). Bone morphogenetic protein (BMP) in the glial scar has shown similar effects (Wang et al., 2011). It has recently been shown that glial scar could benefit in the CNS’s recovery, while previous studies have indicated that glial scar development has adverse effects, such as limiting axonal regeneration (Hernández et al., 2021). By isolating the damaged area from the normal brain, glial scars lessen the likelihood that the inflammatory response will spread across the entire brain. Reactive astrocytes are neuroprotective by limiting the inflammatory response in damaged CNS areas (Sofroniew, 2009). These protective effects include isolation of blood-derived macrophages and repair of the blood–brain barrier. Reactive astrocytes also suppress inflammation by secreting BDNF in the initial stages of glial scar formation (Silver and Miller, 2004; Rolls et al., 2009). It has been reported that the presence of glial scar could be detected a few days after ischemic injury, and mice deficient for glial fibrillary acidic protein and vimentin (GFAP−/−Vim−/−) exhibit less organized and dense glial scar after brain injury, with an infarct area increased, suggesting that glial scar is important in protecting tissue integrity and avoiding further exacerbation of inflammation (Li et al., 2008).

In ischemic stroke, glial scar is a considerable obstacle to neuronal regeneration. Therefore, it is advantageous in stroke to employ strategies that promote their degradation and discourage their formation. miR-124 has been reported to regulate glial scar formation in ischemic stroke. M2 microglial extracellular vesicles could restrict glial scar formation and promote post-stroke recovery by upregulating miR-124 (Li Z. et al., 2021). Consequently, miR-124 might be an essential target for enhancing neuroprotection and recovery in ischemic stroke. Future research for neuroprotection will likely focus on discovering medicines that upregulate miR-124 in ischemic stroke.

Following SCI, glial scar formation is a significant self-defense mechanism (Fitch and Silver, 2008; Sofroniew, 2009; Sofroniew and Vinters, 2010). During the acute phase of SCI, astrocytes adjacent to the injury site are characterized by morphological hypertrophy, increased proliferation, and enhanced expression of the GFAP, Vim, and nestin. All these pathological processes eventually lead to the formation of the glial scar, which has beneficial effects in the acute phase of SCI, especially in repairing the blood-spinal cord barrier and limiting the spread of injury (Okada et al., 2006; Herrmann et al., 2008; Wanner et al., 2013). Certain inhibitory factors secreted by the glial scar in the chronic phase, such as CSPGs, inhibit axonal regeneration. Therefore, coping with glial scar formation at different times of SCI to promote neuroprotection (acute phase) and axonal regeneration (subacute phase and chronic phase) is a potential research direction. It has been discovered that when the Dicer1 gene, which encodes an enzyme required for mature miRNA generation, was conditionally deleted, the injury-induced proliferation of astrocytes was blocked. Synthetic miR-17-5p mimics could rescue the proliferation defect in Dicer1-null astrocytes, while antisense inhibitors of miR-17-5p block LPS-induced astrocyte proliferation (Adams and Gallo, 2018). miR-145 is regarded as a tumor suppressor RNA in several cancer types, such as hepatocellular carcinoma (Noh et al., 2013), ovarian cancer (Kim et al., 2015), and glioma (Iorio et al., 2005). It has been found that healthy rat spinal cord neurons and astrocytes are enriched with miR-145, downregulated in astrocytes 1 week and 1 month after SCI. miR-145 overexpression in astrocytes could reduce astrocyte density at the edge of the damaged spinal cord lesion, inhibit proliferation and migration of reactive astrocytes, and hinder glial scar formation. These findings indicate that miR-145 could prevent spinal cord tissue injury by promoting astrocyte proliferation and glial scar formation (Wang et al., 2015). Similarly, miR-145-5p is a negative regulator of astrocyte proliferation, and its downregulation promotes SMAD3 activity, thereby promoting astrocyte proliferation and glial scar formation (Ye et al., 2022). LncRNAH19 as ceRNA could attenuate the inhibitory effect of miR-1-3p on C-C motif chemokine ligand 2 (CCL2) expression in SCI. The suppression of miR-1-3p could effectively reverse the effects of H19 silencing on normal astrocyte proliferation and activation, suggesting that the H19/miR-1-3p axis regulates astrocyte proliferation and glial scar formation *via* CCL2 (Li P. et al., 2020).

### 2.3. Transcriptomic analysis of reactive astrocytes

Researchers have concluded that differences exist between astrocytes in physiological states due to differences in developmental patterns and extracellular signals (Tsai et al., 2012). The advent of microarray and genome-wide gene expression investigations in recent years has made it clear to researchers that there is heterogeneity among astrocytes, with distinct molecular states in various CNS regions (Itoh et al., 2018) and at different developmental periods (Boisvert et al., 2018). Similarly, following dysregulation of microenvironmental homeostasis, these reactive astrocytes are highly heterogeneous that have both deleterious and beneficial effects (Zhang and Barres, 2010; Zamanian et al., 2012; Liddelow et al., 2017). These heterogeneous responses raise the question of whether there are different subtypes of reactive astrocytes that elicit different responses. Transcriptome analysis of resting and reactive astrocytes isolated from healthy and damaged brains by LPS injection or MCAO identified two distinct types of reactive astrocytes: A1 and A2. Because the different transcriptomic changes of reactive astrocytes bring different pathophysiological changes, more and more studies are now aimed at the transcriptome regulation of reactive astrocytes.

A1 astrocytes, with longer dendrites *in vivo* and *in vitro*, lose the ability to promote neuronal survival, growth, synapse formation, and phagocytosis (Liddelow et al., 2017). In addition, A1 astrocytes secrete a saturated lipid (Guttenplan et al., 2021) that rapidly and selectively kills retinal ganglion cells, cortical neurons, spinal motor neurons, and human dopaminergic neurons, but not preganglionic and gamma motor neurons (Sekar et al., 2016). Oligodendrocytes form and maintain white matter myelin sheaths around axons in the CNS (Morrison et al., 2013). A1 astrocytes conditioned medium could rapidly kill mature differentiated oligodendrocytes and impede the differentiation and maturation of oligodendrocyte precursor cells but do not directly kill oligodendrocyte precursor cells (Liddelow et al., 2017). After LPS injection in neonatal rats, C3aR expression was increased in NG2 oligodendrocyte progenitor cells in the periventricular white matter, and C3a/C3aR signaling might inhibit oligodendrocyte precursor cell differentiation and maturation *via* the Wnt/β-catenin signaling pathway. Thus, it is hypothesized that A1 astrocytes might be responsible for hindering the differentiation of oligodendrocyte precursor cells by releasing C3a, which would lower the amount of axonal myelin in mature periventricular white matter injury (Huang et al., 2020). The complement proteins C1r, C1s, C3, and C4 are upregulated as a result of the recruitment of the IL-1 receptor’s TLR domain by A1 astrocytes *via* the myeloid differentiation factor 88, which also activates NF-κB and MAPK. C3 is now widely acknowledged as a decisive marker of A1 astrocytes. The expression of C3 increases in A1 astrocytes, cleaved into C3a and C3b to trigger downstream events by binding to its receptors C3aR and CR3, respectively (Stephan et al., 2012). Since both C3aR and CR3 are also expressed on microglia, the C3b/CR3 and C3a/C3aR signaling pathways are critical pathways through which A1 astrocytes exert their effects on microglia (Hong et al., 2016; Lian et al., 2016).

In PD models, an increase in A1 astrocytes is frequently accompanied by a decline in dopaminergic neurons (Luna-Herrera et al., 2020). In AD models, the absence of C3 leads to a decline in the expressions of numerous pro-inflammatory factors, including TNF-α, TNF-γ, IL-6, and IL-12, as well as the aging-related loss of synapses and neurons (Shi et al., 2017). Activating microglia CR3 enhances microglia phagocytosis, resulting in reducing synapses in a mouse AD model (Hong et al., 2016). In addition to the C3/CR3 pathway, C3 secreted by A1 astrocytes interacts with microglia C3aR to regulate microglia phagocytosis, β-amyloid, and neuroinflammation in AD models, thereby worsening cognitive function by impairing dendritic cell morphology and synaptic function (Lian et al., 2016; Litvinchuk et al., 2018; Liu et al., 2020).

In chronic cerebral ischemia, the upregulation of C3 expression leads to aberrant microglia activation and promotes microglia redistribution and myelin phagocytosis through activating microglia C3aR, thereby exacerbating brain white matter damage and cognitive dysfunction (Zhang et al., 2020). A1 astrocyte-derived C3 prevents microglia from phagocytosing myelin debris by activating microglia C3aR, which in turn slows myelin redistribution in cerebral hemorrhage (Zheng et al., 2021). The conditioned cultures of A1 astrocytes induced by OGD/R could promote cell apoptosis and reduce the expression of synaptic proteins of cultured cortical neurons, including the scaffolding protein postsynaptic density-95, calmodulin-dependent kinase II, and synaptophysin (Hong et al., 2020). Studies have demonstrated that the neuroprotective effects of intra-arterial selective cooling infusion in hypertensive MCAO rats could be related with phenotype shifting of astrocytes (Wang et al., 2022). Therefore, studies on the neuroprotective effects of miRNAs-regulated A1/A2 astrocytes conversion in ischemic stroke deserve our attention.

The discovery of A1 neurotoxic astrocytes in SCI has provided a new research direction for treating SCI. Neuron-derived exosome-delivered miR-124-3p could reduce neuroinflammation by inhibiting the activation of M1 microglia and A1 astrocytes and promote the recovery of neurological function after SCI (Jiang et al., 2020). The study showed that the expression of miR-21a-5p and the level of A1 marker were up-regulated in spinal cord tissue 3 days after SCI, while the expression of ciliary neurotrophic factor receptor alpha (CNTFRα) was down-regulated. After ciliary neurotrophic factor (CNTF) intervention, A1 marker levels were decreased, while A2 levels were increased. With the downregulation of miR-21a-5p expression, the expression of the A1 marker was significantly reduced, while CNTFRα siRNA intervention had the opposite effects. Therefore, miR-21a-5p might promote A1 astrocyte induction through the downstream target gene CNTFRα and facilitate the inflammatory process of SCI (Zhang et al., 2021). Targeting astrocyte miR-21a-5p is a potential approach to promote SCI rehabilitation in the future.

A2 astrocytes morphologically exhibit hypertrophy with fewer dendrites (Zou et al., 2019). A2 astrocytes release several neurotrophic factors, including arginase-1, NRF2 and platelet-responsive proteins, which promote neuronal survival and synaptic repair (Zamanian et al., 2012; Liddelow and Barres, 2017). Transcriptional profiling of ischemia-induced A2-reactive astrocytes shows upregulation of anti-inflammatory genes such as corticotrophin like cytokine factor 1, S100 calcium-binding protein A10, pentapeptide 3, sphingosine kinase 1, IL-6, leukemia inhibitory factor and transglutaminase 1, as well as the upregulation of neurotrophic factors. On the other hand, A2 astrocytes could activate regulatory factors, including transcription 3, Ras homologous family member A, hypoxia-inducible factor 1α subunit and erythropoietin, which are involved in neural anti-inflammation and neural repair (Liddelow and Barres, 2017; Renault-Mihara et al., 2017; Zhang et al., 2018). Furthermore, numerous investigations have demonstrated an interaction between A2 astrocytes and microglia. The release of TGF-β from A2 astrocytes attenuates microglia activation (Norden et al., 2014). Pentraxin3 (PTX3) is a specific marker of A2 astrocytes. Under inflammatory conditions, both microglia and astrocytes secrete PTX3, which affects the phagocytic activity of microglia (Jeon et al., 2010). In addition to the release of BDNF and vascular endothelial growth factor (VEGF) to promote the differentiation of oligodendrocytes, A2 astrocytes guard against white matter injury by promoting the conversion of oligodendrocyte precursor cells into mature oligodendrocytes through mitochondrial migration. Paradoxically, A2 astrocytes produce prostaglandin E2 through a cyclooxygenase 2-dependent manner, limiting oligodendrocyte precursor cell maturation and myelin formation in a neonatal IL-1β-induced white matter injury model (Shiow et al., 2017). These results suggest that A2 astrocytes may not always benefit oligodendrocyte precursor cell differentiation and development. While in some cases, they may also have deleterious effects.

## 3. Clinical applications of microRNA-targeted regulation of reactive astrocytes

### 3.1. Diagnostic and prognostic indicators

Excitotoxic effects caused by glutamate are the leading contributor of neuronal injury in several neurological diseases. Glutamate transporters on astrocytes are the primary pathway for glutamate uptake. Typically, downregulation of glutamate transporter expression following brain injury leads to a decline in glutamate, aggravating brain injury. In ischemic stroke, miR-107 and miR-124 could worsen or ameliorate brain injury by regulating GLT-1 to promote or inhibit glutamate uptake in astrocytes. miR-107 and miR-124 could be used as new biomarkers to detect the excitotoxicity of glutamate accumulation in ischemic stroke (Yang et al., 2014; Huang et al., 2019). When miR-30a-5p and miR-543-3p bind to SLC1A2 in PD, GLT-1 expression is downregulated, exacerbating the injury. The novel biomarkers miR-30a-5p and miR-543-3p may be utilized to track excitotoxicity in PD (Wu et al., 2019; Meng et al., 2021). In ALS, miR-218 directly silences GLT-1, making astrocytes less able to absorb glutamate. miR-218 is a potential indicator for monitoring glutamate accumulation in ALS (Hoye et al., 2018).

The astrocyte protein AQP4 is pivotal in ischemic brain damage (Zhang et al., 2015). By causing more brain edema, AQP4 exacerbates cerebral ischemia in the rat model of ischemic stroke. The downregulation of AQP4 expression could result from the upregulation of miR-145 and miR-130b, which would lessen brain edema and benefit neurological recovery (Zheng et al., 2017; Wang et al., 2020). According to the above research, miR-145 and miR-130b could be used as vital indicators to determine the severity of cerebral edema during the progression of cerebral ischemia.

After brain injury, the development of the glial scar has a dual impact. In the early stage, Glial scar isolates the spread of inflammation to prevent further injury aggravation. In contrast, the glial scar in the latter stages limits neurological recovery by hindering neural repair and synaptic regeneration. The downregulation of miR-124 expression promotes reactive astrocyte proliferation and glial scar formation in ischemic stroke (Li Z. et al., 2021). miR-124, miR-145-5p, miR-1-3p, and miR-17-5p could regulate glial scar formation in SCI (Hong et al., 2014; Li P. et al., 2020; Li Z. et al., 2021; Ye et al., 2022). miR-124, miR-145-5p, miR-1-3p, and miR-17-5p could be used as indicators of the extent of glial scar formation.

A1 astrocytes of reactive astrocytes after microenvironmental dysregulation in the CNS release inflammatory factors that aggravate brain injury, while A2 astrocytes release neurotrophic factors that play a protective role. The upregulation of miR-136-5p and miR-140 contribute to the release of inflammatory factors in reactive astrocytes in SCI (He et al., 2017; Tu et al., 2017). miR-124-3p and miR-21a-5p promote the transformation of astrocytes to A1 astrocytes (Jiang et al., 2020; Zhang et al., 2021). As a result, the level of inflammatory activity in SCI might be assessed using miR-136-5p, miR-140, miR-124-3p, and miR-21a-5p.

**Figure 2:**
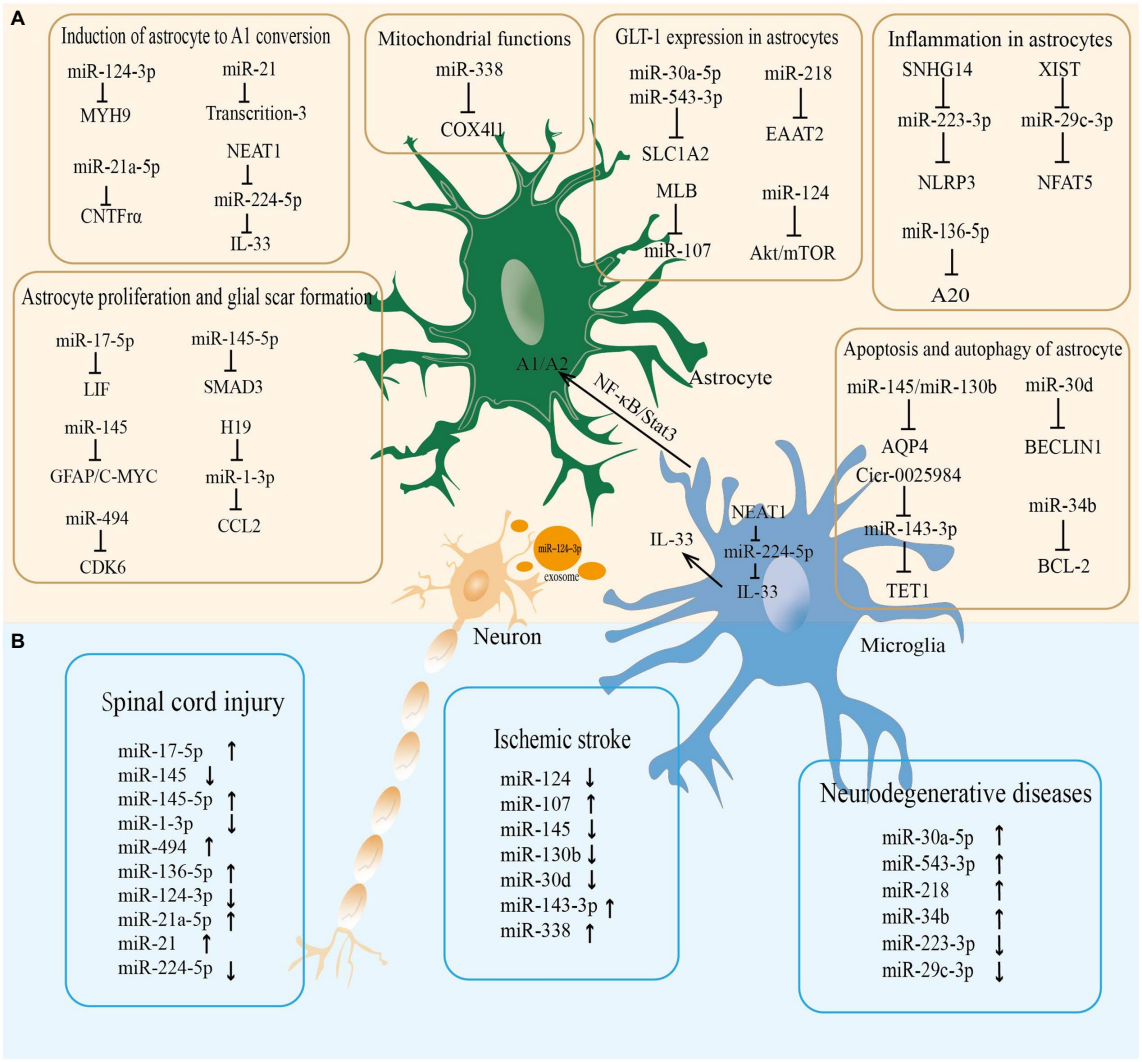
The miRNA-mRNA regulatory network in astrocytes regulated neurological diseases. **(A)** The microRNAs regulate the pathophysiological functions of astrocytes in the central nervous system. **(B)** The microRNAs altered in neurological diseases.

miR-338 is involved in regulating mitochondrial oxygen consumption, ATP production, and ROS production in axons in the CNS (Aschrafi et al., 2012). After 20 min of whole brain ischemia and 30 min of reperfusion in rats, miR-338 levels are upregulated more than 2-fold in the hippocampus of rats (Di et al., 2014), which is considerably raised in the cerebrospinal fluid (CSF) of patients with subacute ischemic stroke (Peng et al., 2015). As a highly sensitive biomarker of mitochondrial toxicity, miR-338 has been employed (Baumgart et al., 2016). MiR-338 can promote the production of COX IV and the mitochondrial ATP synthase ATP5G1 subunit, which significantly affects neuronal ROS levels and axonal development (Aschrafi et al., 2012; Figure 2; Table 1).

**Table 1:** Characteristics of miRNA involved in astrocytes in Neurological disorders.

microRNA	Disorders	Up/Downregulation	Target genes	Upstream	Influence on astrocytes
miR-17-5p	SCI	Up	LIF		Promotion of astrocyte proliferation and glial scar formation
miR-145	SCI	Down	GFAP/C-MYC		Promotion of astrocyte proliferation and glial scar formation
miR-145-5p	SCI	Up	SMAD3		Promotion of astrocyte proliferation and glial scar formation
miR-1-3p	SCI	Down	CCL2	H19	Promotion of astrocyte proliferation and glial scar formation
miR-124	Ischemic stroke	Down	STAT3		Promotion of astrocyte proliferation and glial scar formation
miR-494	SCI	Up	CDK6		Inhibition of astrocyte proliferation and synaptic remodeling
miR-107	Ischemic stroke	Up		MLB	Inhibition of GLT-1 expression in astrocytes
miR-124	Ischemic stroke	Down	AKT/mTOR		Inhibition of GLT-1 expression in astrocytes
miR-30a-5p	PD	Up	SLC1A2		Promotion of GLT-1 degradation in astrocytes
miR-543-3p	PD	Up	SLC1A2		Inhibition of GLT-1 expression in astrocytes
miR-218	ALS	Up	EAAT2		Astrocyte dysfunction
miR-145	Ischemic stroke	Down	AQP4		Promotion of apoptosis of astrocytes
miR-130b	Ischemic stroke	Down	AQP4		Promotion of apoptosis of astrocytes
miR-30d	Ischemic stroke	Down	BECLIN1		Promotion of autophagy in astrocytes and inhibition of apoptosis in astrocytes
miR-143-3p	Ischemic stroke	Up	TET1	Circ_0025984	Promotion of apoptosis and autophagy in astrocytes
miR-34b	Epilepsy	Up	BCL-2		Promotion of apoptosis in astrocyte
miR-338	Ischemic stroke	Up	COX4l1		Inhibition of Mitochondrial function
miR-136-5p	SCI	Up	A20		Promotion of astrocyte activation and inflammatory factor release
miR-223-3p	AD	Down	NLRP3	SNHG14	Promotion of neuroinflammation development
miR-29c-3p	Epilepsy	Down	NFAT5	XIST	Promotion of astrocyte activation and inflammatory factor release
miR-124-3p	SCI	Down	MYH9		Induction of astrocyte to A1 conversion
miR-21a-5p	SCI	Up	CNTFrα		Induction of astrocyte to A1 conversion
miR-21	SCI	Up	Transcription-3		Induction of A1 to A2 conversion
miR-224-5p	SCI	Down	IL-33	NEAT1	Inhibition of Induction of astrocyte to A1 conversion

SCI, spinal cord injury; PD, Parkinson’s disease; ALS, amyotrophic lateral sclerosis; LIF, leukemia inhibitory factor; GFAP, glial fibrillary acidic protein; C-MYC, MYC proto-oncogene, bHLH transcription factor; SMAD3, SMAD family member 3; CCL2, C-C motif chemokine ligand 2; H19, H19 imprinted maternally expressed transcript; STAT3, signal transducer and activator of transcription 3; CDK6, cyclin dependent kinase 6; MLB, magnesium lithospermate B; AKT, AKT serine/threonine kinase 1; mTOR, mechanistic target of rapamycin kinase; SLC1A2, solute carrier family 1 member 2; EAAT2, excitatory amino acid transporter 2; AQP4, aquaporin 4; TET1, tet methylcytosine dioxygenase 1; BCL2, BCL2 apoptosis regulator; COX4l1, cytochrome c oxidase subunit 4I1 pseudogene 1; NLRP3, NLR family pyrin domain containing 3; SNHG14,small nucleolar RNA host gene 14; NFAT5, nuclear factor of activated T cells 5; XIST, X inactive specific transcript; MYH9, myosin heavy chain 9; CNTFrα, ciliary neurotrophic factor receptor α; IL-33, interleukin 33; NEAT1, nuclear paraspeckle assembly transcript 1.

### 3.2. Therapeutic targets

How to translate the regulatory effects of miRNAs on astrocytes in neurological disorders into clinically meaningful effects is a question that deserves our consideration. miRNA-based therapies could be divided into two different approaches: (1) the use of miRNA antagonists (antisense oligonucleotides, counterparts, and miRNA sponges) or drugs targeting miRNAs to inhibit the adverse effects of miRNAs in neurological diseases, and (2) the use of miRNA mimics, exosome-carried miRNAs or knockdown of miRNA sponges to upregulate the expression of miRNAs.

The upregulation of miR-143-3p could promote astrocyte apoptosis and autophagy *via* TET1/ORP150, exacerbating brain injury in cerebral ischemia. miR-143-3p inhibitor and circ_0025984 (a miR-143-3p sponge) have been found to drastically limit astrocyte apoptosis and autophagy, mitigating brain injury and neuronal loss in ischemic stroke (Zhou et al., 2021). Certain studies have reported that a few drugs target miRNAs to affect the activity and function of astrocytes to benefit the recovery of neurological functions in neurological diseases. For instance, MLB plays a neuronal protective role and promotes functional recovery after stroke by inhibiting the upregulation of miR-107 on astrocyte GLT-1 (Yang et al., 2015). Ang-(1–7) is a member of the renin-angiotensin system (RAS) and is produced by Angiotensin II (Ang II). Ang-(1–7) promotes functional recovery after stroke by binding to the MAS1 receptor, which is influential in the pathogenesis of several neurological disorders such as AD (Xu et al., 2011). Ang-(1–7) exerts a neuroprotective effect by inhibiting neuroinflammation mediated by astrocyte NLRP3 inflammasome through the SNHG14/miR-223-3p/NLRP3 pathway in AD (Duan et al., 2021).

The downregulation of miR-130b in astrocytes leads to the upregulation of water channel protein AQP4 and exacerbates brain injury in cerebral ischemia. miR-130b mimics could exert neuroprotective effects by decreasing the expression of AQP4 on astrocyte membranes and alleviating ischemia-induced damage (Zheng et al., 2017). Bone marrow mesenchymal stem cells (BMSCs)-carried miR-146a suppresses the release of inflammatory factors from reactive astrocytes and improves cognitive impairment in AD models (Nakano et al., 2020). In a study of depression, miR-207 is downregulated in brain tissue, weakens the inhibitory effect on TRIL, and facilitates the release of inflammatory factors in reactive astrocytes. NK cell-derived exosomes could upregulate miR-207 expression, inhibit the release of inflammatory factors in reactive astrocytes, and alleviate chronic mild stress symptoms in mice (Li D. et al., 2020). In a mouse model of tMCAO, circHECTD1, an endogenous miR-142 sponge, could inhibit miR-142 activity to downregulate TIPARP (TCDD-inducible poly [ADP-ribose] polymerase) expression, impede astrocyte autophagy, and ultimately increase infarct size. The upregulation of miR-142 by knocking down circHECTD1 drives astrocyte autophagy and shrinks infarct size (Han et al., 2018). CircHIPK2, an endogenous miR-124-2HG sponge, upregulates sigma non-opioid intracellular receptor 1 (SIGMAR1/OPRS1) expression, promotes astrocyte autophagy and neuroinflammatory responses, and finally aggravates brain tissue damage. Inhibiting circHIPK2 expression could upregulate miR-142 expression to suppress astrocyte autophagy and endoplasmic reticulum stress, preventing brain tissue damage from neuroinflammatory (Huang et al., 2017). The upregulation of miR-145 by knocking down LncRNA MALAT1 could inhibit AQP4 expression to improve brain I/R injury (Wang et al., 2020). LncRNA MEG exacerbates neuropathic pain and astrocyte activation through miR-130a-5p/CXCL12/CXCR4 axis. The pro-inflammatory effect of miR-130a-5p on reactive astrocytes is attenuated by silencing MEG3 to relieve neuropathic pain (Dong et al., 2021).

## 4. Discussion and conclusion

The CNS microenvironment’s homeostasis is crucially maintained by astrocytes. Reactive astrocytes perform imperative pathophysiological functions in CNS diseases such as stroke, SCI, and neurodegenerative disease. miRNAs are non-coding RNAs involved in neuronal cell behavior and nervous system development. miRNAs play a positive or negative role in astrocyte-mediated neurological diseases. The regulatory mechanisms of miRNAs are related to the transcriptomic alterations, morphology, and function of reactive astrocytes. The current study has found that regulating the miRNAs-astroglial axis could inhibit the pathophysiologic progression of CNS diseases.

The current research findings indicate that miRNA-mediated regulation of reactive astrocytes in CNS diseases is a potential research direction to promote neurological recovery. However, significant obstacles must yet be overcome. (1) One is the specificity of miRNA-based therapeutics. Each miRNA has dozens of potential targets. MiRNAs have a distinct advantage in controlling intricate biological processes due to their capacity to control numerous mRNAs. It could be difficult to target miRNAs to exert protective effects on specific targets in CNS disorders. Target site blockers (TSBs) are crucial for preventing miRNAs from regulating specific mRNAs and identifying specific miRNA-mRNA interaction networks. However, the design principles of TSBs are not yet fully understood and remain a challenge. (2) The second is that astrocytes are highly heterogeneous glial cells whose transcriptomic changes, morphology, and functions are highly heterogeneous not only in different brain regions but also at different time stages after CNS injury. Whether miRNAs have different effects on other functions of the same astrocytes or on the functions of astrocytes at different time stages requires further exploration. (3) Third, How to efficiently transport miRNAs to the CNS through the blood–brain barrier to exert neuroprotective effects. miRNA mimics and inhibitors enable miRNAs to exist in circulation for longer, but most miRNAs accumulate in the liver and kidney and cannot be efficiently transported to the CNS. Therefore, it is essential to explore the ways for the targeted transport of miRNAs to the CNS. Viral and non-viral delivery systems specific to brain endothelial cells have been found to deliver nucleotide-based drugs to the brain (Marcos-Contreras et al., 2020). But it remains to be verified whether this mode of drug delivery is toxic and whether immune rejection occurs. Exosome, an important miRNA carrier discovered in recent years, is characterized by high stability, easy passage through the blood–brain barrier, and little graft response. The miRNAs-exosome targeting astrocytes is a promising treatment for neurological diseases.

In conclusion, studies based on the miRNAs-astrocyte axis are currently inadequate in CNS diseases. Focusing on the role and function of miRNAs in regulating reactive astrocytes, we summarize the regulatory mechanism of miRNAs on reactive astrocytes, which might provide a theoretical basis for the diagnosis and treatment of CNS diseases.

## Author contributions

All authors listed have made a substantial, direct, and intellectual contribution to the work and approved it for publication.

## Funding

This research was supported by grants from the National Natural Science Foundation of China (81701216 and 82172955), Wuxi Taihu Lake Talent Plan, Supports for Leading Talents in Medical and Health Profession (2020THRC-DJ-SNW), Natural Science Foundation of Jiangsu Province (BK20160197 and BK20180169), and Reserve Talents of Double Hundred Talent Plan (HB2020021).

## Conflict of interest

The authors declare that the research was conducted in the absence of any commercial or financial relationships that could be construed as a potential conflict of interest.

## Publisher’s note

All claims expressed in this article are solely those of the authors and do not necessarily represent those of their affiliated organizations, or those of the publisher, the editors and the reviewers. Any product that may be evaluated in this article, or claim that may be made by its manufacturer, is not guaranteed or endorsed by the publisher.

## References

[ref1] Adams K. L. Gallo V. (2018). The diversity and disparity of the glial scar. Nat. Neurosci. 21, 9–15. doi: 10.1038/s41593-017-0033-9 29269757 PMC5937232

[ref2] Aguirre-Gamboa R. Joosten I. Urbano P. van der Molen R. G. van Rijssen E. van Cranenbroek B.. (2016). Differential effects of environmental and genetic factors on T and B cell immune traits. Cell Rep. 17, 2474–2487. doi: 10.1016/j.celrep.2016.10.053, PMID: 27818087 PMC5130901

[ref3] Ahuja C. S. Nori S. Tetreault L. Wilson J. Kwon B. Harrop J.. (2017). Traumatic spinal cord injury-repair and regeneration. Neurosurgery 80, S9–S22. doi: 10.1093/neuros/nyw080, PMID: 28350947

[ref4] Al-Chalabi A. Hardiman O. (2013). The epidemiology of ALS: a conspiracy of genes, environment and time. Nat. Rev. Neurol. 9, 617–628. doi: 10.1038/nrneurol.2013.203, PMID: 24126629

[ref5] Allaman I. Bélanger M. Magistretti P. J. (2011). Astrocyte-neuron metabolic relationships: for better and for worse. Trends Neurosci. 34, 76–87. doi: 10.1016/j.tins.2010.12.001, PMID: 21236501

[ref6] Allen N. J. Eroglu C. (2017). Cell biology of astrocyte-synapse interactions. Neuron 96, 697–708. doi: 10.1016/j.neuron.2017.09.056, PMID: 29096081 PMC5687890

[ref7] Alvarez J. I. Katayama T. Prat A. (2013). Glial influence on the blood brain barrier. Glia 61, 1939–1958. doi: 10.1002/glia.22575, PMID: 24123158 PMC4068281

[ref8] Ambrosi G. Cerri S. Blandini F. (2014). A further update on the role of excitotoxicity in the pathogenesis of Parkinson's disease. J. Neural Transm. (Vienna) 121, 849–859. doi: 10.1007/s00702-013-1149-z, PMID: 24380931

[ref9] Andersen J. V. Jakobsen E. Westi E. W. Lie M. Voss C. M. Aldana B. I.. (2020). Extensive astrocyte metabolism of γ-aminobutyric acid (GABA) sustains glutamine synthesis in the mammalian cerebral cortex. Glia 68, 2601–2612. doi: 10.1002/glia.23872, PMID: 32584476

[ref10] Arriza J. L. Eliasof S. Kavanaugh M. P. Amara S. G. (1997). Excitatory amino acid transporter 5, a retinal glutamate transporter coupled to a chloride conductance. Proc. Natl. Acad. Sci. U. S. A. 94, 4155–4160. doi: 10.1073/pnas.94.8.4155, PMID: 9108121 PMC20584

[ref11] Arriza J. L. Fairman W. A. Wadiche J. I. Murdoch G. H. Kavanaugh M. P. Amara S. G. (1994). Functional comparisons of three glutamate transporter subtypes cloned from human motor cortex. J. Neurosci. 14, 5559–5569. doi: 10.1523/JNEUROSCI.14-09-05559.1994, PMID: 7521911 PMC6577102

[ref12] Aschrafi A. Kar A. N. Natera-Naranjo O. Mac Gibeny M. A. Gioio A. E. Kaplan B. B. (2012). Micro RNA-338 regulates the axonal expression of multiple nuclear-encoded mitochondrial mRNAs encoding subunits of the oxidative phosphorylation machinery. Cell. Mol. Life Sci. 69, 4017–4027. doi: 10.1007/s00018-012-1064-8, PMID: 22773120 PMC11114659

[ref13] Bartel D. P. (2004). MicroRNAs: genomics, biogenesis, mechanism, and function. Cells 116, 281–297. doi: 10.1016/s0092-8674(04)00045-5 14744438

[ref14] Baumgart B. R. Gray K. L. Woicke J. Bunch R. T. Sanderson T. P. Van Vleet T. R. (2016). MicroRNA as biomarkers of mitochondrial toxicity. Toxicol. Appl. Pharmacol. 312, 26–33. doi: 10.1016/j.taap.2015.10.007 26476301

[ref15] Boisvert M. M. Erikson G. A. Shokhirev M. N. Allen N. J. (2018). The aging astrocyte Transcriptome from multiple regions of the mouse brain. Cell Rep. 22, 269–285. doi: 10.1016/j.celrep.2017.12.039, PMID: 29298427 PMC5783200

[ref16] Boulay A. C. Cisternino S. Cohen-Salmon M. (2016). Immunoregulation at the gliovascular unit in the healthy brain: a focus on Connexin 43. Brain Behav. Immun. 56, 1–9. doi: 10.1016/j.bbi.2015.11.017, PMID: 26674996

[ref17] Bradbury E. J. Carter L. M. (2011). Manipulating the glial scar: chondroitinase ABC as a therapy for spinal cord injury. Brain Res. Bull. 84, 306–316. doi: 10.1016/j.brainresbull.2010.06.015, PMID: 20620201

[ref18] Brod S. A. (2022). Anti-inflammatory agents: an approach to prevent cognitive decline in Alzheimer's disease. J. Alzheimers Dis. 85, 457–472. doi: 10.3233/JAD-215125, PMID: 34842189

[ref19] Cassina P. Cassina A. Pehar M. Castellanos R. Gandelman M. de León A.. (2008). Mitochondrial dysfunction in SOD1G93A-bearing astrocytes promotes motor neuron degeneration: prevention by mitochondrial-targeted antioxidants. J. Neurosci. 28, 4115–4122. doi: 10.1523/JNEUROSCI.5308-07.2008, PMID: 18417691 PMC3844766

[ref20] Chang C. H. Lin C. H. Lane H. Y. (2020). D-glutamate and gut microbiota in Alzheimer's disease. Int. J. Mol. Sci. 21:2676. doi: 10.3390/ijms21082676, PMID: 32290475 PMC7215955

[ref21] Chang T. C. Yu D. Lee Y. S. Wentzel E. A. Arking D. E. West K. M.. (2008). Widespread microRNA repression by Myc contributes to tumorigenesis. Nat. Genet. 40, 43–50. doi: 10.1038/ng.2007.30, PMID: 18066065 PMC2628762

[ref22] Chisholm N. C. Henderson M. L. Selvamani A. Park M. J. Dindot S. Miranda R. C.. (2015). Histone methylation patterns in astrocytes are influenced by age following ischemia. Epigenetics 10, 142–152. doi: 10.1080/15592294.2014.1001219, PMID: 25565250 PMC4622874

[ref23] Cobley J. N. Fiorello M. L. Bailey D. M. (2018). 13 reasons why the brain is susceptible to oxidative stress. Redox Biol. 15, 490–503. doi: 10.1016/j.redox.2018.01.008, PMID: 29413961 PMC5881419

[ref24] Conti F. Minelli A. Melone M. (2004). GABA transporters in the mammalian cerebral cortex: localization, development and pathological implications. Brain Res. Brain Res. Rev. 45, 196–212. doi: 10.1016/j.brainresrev.2004.03.003, PMID: 15210304

[ref25] Cua R. C. Lau L. W. Keough M. B. Midha R. Apte S. S. Yong V. W. (2013). Overcoming neurite-inhibitory chondroitin sulfate proteoglycans in the astrocyte matrix. Glia 61, 972–984. doi: 10.1002/glia.22489, PMID: 23554135

[ref26] Daneman R. Engelhardt B. (2017). Brain barriers in health and disease. Neurobiol. Dis. 107, 1–3. doi: 10.1016/j.nbd.2017.05.008 28552387

[ref27] Di Y. Lei Y. Yu F. Changfeng F. Song W. Xuming M. (2014). MicroRNAs expression and function in cerebral ischemia reperfusion injury. J. Mol. Neurosci. 53, 242–250. doi: 10.1007/s12031-014-0293-8 24696166

[ref28] Dickendesher T. L. Baldwin K. T. Mironova Y. A. Koriyama Y. Raiker S. J. Askew K. L.. (2012). NgR1 and NgR3 are receptors for chondroitin sulfate proteoglycans. Nat. Neurosci. 15, 703–712. doi: 10.1038/nn.3070, PMID: 22406547 PMC3337880

[ref29] Dong J. Xia R. Zhang Z. Xu C. (2021). lncRNA MEG3 aggravated neuropathic pain and astrocyte overaction through mediating miR-130a-5p/CXCL12/CXCR4 axis. Aging (Albany NY) 13, 23004–23019. doi: 10.18632/aging.203592, PMID: 34609952 PMC8544300

[ref30] Duan R. Wang S. Y. Wei B. Deng Y. Fu X. X. Gong P. Y.. (2021). Angiotensin-(1-7) analogue AVE0991 modulates astrocyte-mediated Neuroinflammation via lncRNA SNHG14/miR-223-3p/NLRP3 pathway and offers Neuroprotection in a transgenic mouse model of Alzheimer's disease. J. Inflamm. Res. 14, 7007–7019. doi: 10.2147/JIR.S343575, PMID: 34955647 PMC8694579

[ref31] Dyck S. M. Karimi-Abdolrezaee S. (2015). Chondroitin sulfate proteoglycans: key modulators in the developing and pathologic central nervous system. Exp. Neurol. 269, 169–187. doi: 10.1016/j.expneurol.2015.04.006, PMID: 25900055

[ref32] Dzamba D. Honsa P. Valny M. Kriska J. Valihrach L. Novosadova V.. (2015). Quantitative analysis of glutamate receptors in glial cells from the cortex of GFAP/EGFP mice following ischemic injury: focus on NMDA receptors. Cell. Mol. Neurobiol. 35, 1187–1202. doi: 10.1007/s10571-015-0212-8, PMID: 25994914 PMC11486180

[ref33] Fairman W. A. Vandenberg R. J. Arriza J. L. Kavanaugh M. P. Amara S. G. (1995). An excitatory amino-acid transporter with properties of a ligand-gated chloride channel. Nature 375, 599–603. doi: 10.1038/375599a0 7791878

[ref34] Ferraiuolo L. Higginbottom A. Heath P. R. Barber S. Greenald D. Kirby J.. (2011). Dysregulation of astrocyte-motoneuron cross-talk in mutant superoxide dismutase 1-related amyotrophic lateral sclerosis. Brain 134, 2627–2641. doi: 10.1093/brain/awr193, PMID: 21908873 PMC3170534

[ref35] Fitch M. T. Silver J. (2008). CNS injury, glial scars, and inflammation: inhibitory extracellular matrices and regeneration failure. Exp. Neurol. 209, 294–301. doi: 10.1016/j.expneurol.2007.05.014, PMID: 17617407 PMC2268907

[ref36] Fleming J. C. Norenberg M. D. Ramsay D. A. Dekaban G. A. Marcillo A. E. Saenz A. D.. (2006). The cellular inflammatory response in human spinal cords after injury. Brain 129, 3249–3269. doi: 10.1093/brain/awl296 17071951

[ref37] Fricker M. Tolkovsky A. M. Borutaite V. Coleman M. Brown G. C. (2018). Neuronal cell death. Physiol. Rev. 98, 813–880. doi: 10.1152/physrev.00011.2017, PMID: 29488822 PMC5966715

[ref38] Fukuda A. M. Badaut J. (2012). Aquaporin 4: a player in cerebral edema and neuroinflammation. J. Neuroinflammation 9:279. doi: 10.1186/1742-2094-9-279, PMID: 23270503 PMC3552817

[ref39] Gardoni F. Di Luca M. (2015). Targeting glutamatergic synapses in Parkinson's disease. Curr. Opin. Pharmacol. 20, 24–28. doi: 10.1016/j.coph.2014.10.011 25462288

[ref40] Guérit S. Fidan E. Macas J. Czupalla C. J. Figueiredo R. Vijikumar A.. (2021). Astrocyte-derived Wnt growth factors are required for endothelial blood-brain barrier maintenance. Prog. Neurobiol. 199:101937. doi: 10.1016/j.pneurobio.2020.101937, PMID: 33383106

[ref41] Guttenplan K. A. Weigel M. K. Prakash P. Wijewardhane P. R. Hasel P. Rufen-Blanchette U.. (2021). Neurotoxic reactive astrocytes induce cell death via saturated lipids. Nature 599, 102–107. doi: 10.1038/s41586-021-03960-y, PMID: 34616039 PMC12054010

[ref42] Haley M. J. Lawrence C. B. (2017). The blood-brain barrier after stroke: structural studies and the role of transcytotic vesicles. J. Cereb. Blood Flow Metab. 37, 456–470. doi: 10.1177/0271678X16629976, PMID: 26823471 PMC5322831

[ref43] Hamilton N. B. Attwell D. (2010). Do astrocytes really exocytose neurotransmitters. Nat. Rev. Neurosci. 11, 227–238. doi: 10.1038/nrn2803, PMID: 20300101

[ref44] Hammond T. R. Gadea A. Dupree J. Kerninon C. Nait-Oumesmar B. Aguirre A.. (2014). Astrocyte-derived endothelin-1 inhibits remyelination through notch activation. Neuron 81, 588–602. doi: 10.1016/j.neuron.2013.11.015, PMID: 24507193 PMC3935216

[ref45] Hammond T. R. McEllin B. Morton P. D. Raymond M. Dupree J. Gallo V. (2015). Endothelin-B receptor activation in astrocytes regulates the rate of Oligodendrocyte regeneration during Remyelination. Cell Rep. 13, 2090–2097. doi: 10.1016/j.celrep.2015.11.002, PMID: 26628380

[ref46] Han B. Zhang Y. Zhang Y. Bai Y. Chen X. Huang R.. (2018). Novel insight into circular RNA HECTD1 in astrocyte activation via autophagy by targeting MIR142-TIPARP: implications for cerebral ischemic stroke. Autophagy 14, 1164–1184. doi: 10.1080/15548627.2018.1458173, PMID: 29938598 PMC6103660

[ref47] Harvey B. K. Airavaara M. Hinzman J. Wires E. M. Chiocco M. J. Howard D. B.. (2011). Targeted over-expression of glutamate transporter 1 (GLT-1) reduces ischemic brain injury in a rat model of stroke. PLoS One 6:e22135. doi: 10.1371/journal.pone.0022135, PMID: 21853027 PMC3154194

[ref48] Hasel P. Rose I. Sadick J. S. Kim R. D. Liddelow S. A. (2021). Neuroinflammatory astrocyte subtypes in the mouse brain. Nat. Neurosci. 24, 1475–1487. doi: 10.1038/s41593-021-00905-6, PMID: 34413515

[ref49] He Y. Liu X. Chen Z. (2020). Glial scar-a promising target for improving outcomes after CNS injury. J. Mol. Neurosci. 70, 340–352. doi: 10.1007/s12031-019-01417-6, PMID: 31776856

[ref50] He J. Zhao J. Peng X. Shi X. Zong S. Zeng G. (2017). Molecular mechanism of MiR-136-5p targeting NF-κB/A20 in the IL-17-mediated inflammatory response after spinal cord injury. Cell. Physiol. Biochem. 44, 1224–1241. doi: 10.1159/000485452, PMID: 29179211

[ref51] Heneka M. T. Kummer M. P. Stutz A. Delekate A. Schwartz S. Vieira-Saecker A.. (2013). NLRP3 is activated in Alzheimer's disease and contributes to pathology in APP/PS1 mice. Nature 493, 674–678. doi: 10.1038/nature11729, PMID: 23254930 PMC3812809

[ref52] Hernández I. H. Villa-González M. Martín G. Soto M. Pérez-Álvarez M. J. (2021). Glial cells as therapeutic approaches in brain ischemia-reperfusion injury. Cells 10:1639. doi: 10.3390/cells10071639, PMID: 34208834 PMC8305833

[ref53] Herrmann J. E. Imura T. Song B. Qi J. Ao Y. Nguyen T. K.. (2008). STAT3 is a critical regulator of astrogliosis and scar formation after spinal cord injury. J. Neurosci. 28, 7231–7243. doi: 10.1523/JNEUROSCI.1709-08.2008, PMID: 18614693 PMC2583788

[ref54] Hirsch E. C. Hunot S. (2009). Neuroinflammation in Parkinson's disease: a target for neuroprotection. Lancet Neurol. 8, 382–397. doi: 10.1016/S1474-4422(09)70062-6 19296921

[ref55] Hong S. Beja-Glasser V. F. Nfonoyim B. M. Frouin A. Li S. Ramakrishnan S.. (2016). Complement and microglia mediate early synapse loss in Alzheimer mouse models. Science 352, 712–716. doi: 10.1126/science.aad8373, PMID: 27033548 PMC5094372

[ref56] Hong P. Jiang M. Li H. (2014). Functional requirement of dicer 1 and miR-17-5p in reactive astrocyte proliferation after spinal cord injury in the mouse. Glia 62, 2044–2060. doi: 10.1002/glia.22725, PMID: 25043492

[ref57] Hong Y. Liu Q. Peng M. Bai M. Li J. Sun R.. (2020). High-frequency repetitive transcranial magnetic stimulation improves functional recovery by inhibiting neurotoxic polarization of astrocytes in ischemic rats. J. Neuroinflammation 17:150. doi: 10.1186/s12974-020-01747-y, PMID: 32375835 PMC7203826

[ref58] Hoye M. L. Regan M. R. Jensen L. A. Lake A. M. Reddy L. V. Vidensky S.. (2018). Motor neuron-derived microRNAs cause astrocyte dysfunction in amyotrophic lateral sclerosis. Brain 141, 2561–2575. doi: 10.1093/brain/awy182, PMID: 30007309 PMC6113638

[ref59] Huang W. Y. Jiang C. Ye H. B. Jiao J. T. Cheng C. Huang J.. (2019). miR-124 upregulates astrocytic glutamate transporter-1 via the Akt and mTOR signaling pathway post ischemic stroke. Brain Res. Bull. 149, 231–239. doi: 10.1016/j.brainresbull.2019.04.013, PMID: 31004734

[ref60] Huang R. Zhang Y. Han B. Bai Y. Zhou R. Gan G.. (2017). Circular RNA HIPK2 regulates astrocyte activation via cooperation of autophagy and ER stress by targeting MIR124-2HG. Autophagy 13, 1722–1741. doi: 10.1080/15548627.2017.1356975, PMID: 28786753 PMC5640207

[ref61] Huang P. Zhou Q. Lin Q. Lin L. Wang H. Chen X.. (2020). Complement C3a induces axonal hypomyelination in the periventricular white matter through activation of WNT/β-catenin signal pathway in septic neonatal rats experimentally induced by lipopolysaccharide. Brain Pathol. 30, 495–514. doi: 10.1111/bpa.12798, PMID: 31622511 PMC8018074

[ref62] Iadecola C. Nedergaard M. (2007). Glial regulation of the cerebral microvasculature. Nat. Neurosci. 10, 1369–1376. doi: 10.1038/nn2003 17965657

[ref63] Iorio M. V. Ferracin M. Liu C. G. Veronese A. Spizzo R. Sabbioni S.. (2005). MicroRNA gene expression deregulation in human breast cancer. Cancer Res. 65, 7065–7070. doi: 10.1158/0008-5472.CAN-05-1783 16103053

[ref64] Itoh N. Itoh Y. Tassoni A. Ren E. Kaito M. Ohno A.. (2018). Cell-specific and region-specific transcriptomics in the multiple sclerosis model: focus on astrocytes. Proc. Natl. Acad. Sci. U. S. A. 115, E302–E309. doi: 10.1073/pnas.1716032115, PMID: 29279367 PMC5777065

[ref65] Jackson R. J. Meltzer J. C. Nguyen H. Commins C. Bennett R. E. Hudry E.. (2022). APOE4 derived from astrocytes leads to blood-brain barrier impairment. Brain 145, 3582–3593. doi: 10.1093/brain/awab478, PMID: 34957486 PMC9586546

[ref66] Jeon H. Lee S. Lee W. H. Suk K. (2010). Analysis of glial secretome: the long pentraxin PTX3 modulates phagocytic activity of microglia. J. Neuroimmunol. 229, 63–72. doi: 10.1016/j.jneuroim.2010.07.001, PMID: 20674043

[ref67] Jiang D. Gong F. Ge X. Lv C. Huang C. Feng S.. (2020). Neuron-derived exosomes-transmitted miR-124-3p protect traumatically injured spinal cord by suppressing the activation of neurotoxic microglia and astrocytes. J Nanobiotechnol. 18:105. doi: 10.1186/s12951-020-00665-8, PMID: 32711535 PMC7382861

[ref68] Jimenez-Blasco D. Santofimia-Castaño P. Gonzalez A. Almeida A. Bolaños J. P. (2015). Astrocyte NMDA receptors' activity sustains neuronal survival through a Cdk5-Nrf2 pathway. Cell Death Differ. 22, 1877–1889. doi: 10.1038/cdd.2015.49, PMID: 25909891 PMC4648333

[ref69] Khaing Z. Z. Milman B. D. Vanscoy J. E. Seidlits S. K. Grill R. J. Schmidt C. E. (2011). High molecular weight hyaluronic acid limits astrocyte activation and scar formation after spinal cord injury. J. Neural Eng. 8:046033. doi: 10.1088/1741-2560/8/4/046033, PMID: 21753237

[ref70] Kim T. H. Song J. Y. Park H. Jeong J. Y. Kwon A. Y. Heo J. H.. (2015). miR-145, targeting high-mobility group A2, is a powerful predictor of patient outcome in ovarian carcinoma. Cancer Lett. 356, 937–945. doi: 10.1016/j.canlet.2014.11.011, PMID: 25444913

[ref71] Kitchen P. Salman M. M. Halsey A. M. Clarke-Bland C. MacDonald J. A. Ishida H.. (2020). Targeting Aquaporin-4 subcellular localization to treat central nervous system edema. Cells 181, 784–799.e19. doi: 10.1016/j.cell.2020.03.037, PMID: 32413299 PMC7242911

[ref72] Krebs C. Fernandes H. B. Sheldon C. Raymond L. A. Baimbridge K. G. (2003). Functional NMDA receptor subtype 2B is expressed in astrocytes after ischemia in vivo and anoxia in vitro. J. Neurosci. 23, 3364–3372. doi: 10.1523/JNEUROSCI.23-08-03364.2003, PMID: 12716944 PMC6742326

[ref73] Kwon H. S. Koh S. H. (2020). Neuroinflammation in neurodegenerative disorders: the roles of microglia and astrocytes. Transl Neurodegener 9:42. doi: 10.1186/s40035-020-00221-2, PMID: 33239064 PMC7689983

[ref74] Lanfranco M. F. Sepulveda J. Kopetsky G. Rebeck G. W. (2021). Expression and secretion of apoE isoforms in astrocytes and microglia during inflammation. Glia 69, 1478–1493. doi: 10.1002/glia.23974, PMID: 33556209 PMC8717762

[ref75] Lanjakornsiripan D. Pior B. J. Kawaguchi D. Furutachi S. Tahara T. Katsuyama Y.. (2018). Layer-specific morphological and molecular differences in neocortical astrocytes and their dependence on neuronal layers. Nat. Commun. 9:1623. doi: 10.1038/s41467-018-03940-3, PMID: 29691400 PMC5915416

[ref76] Li P. Li Y. Dai Y. Wang B. Li L. Jiang B.. (2020). The LncRNA H19/miR-1-3p/CCL2 axis modulates lipopolysaccharide (LPS) stimulation-induced normal human astrocyte proliferation and activation. Cytokine 131:155106. doi: 10.1016/j.cyto.2020.155106, PMID: 32371379

[ref77] Li L. Lundkvist A. Andersson D. Wilhelmsson U. Nagai N. Pardo A. C.. (2008). Protective role of reactive astrocytes in brain ischemia. J. Cereb. Blood Flow Metab. 28, 468–481. doi: 10.1038/sj.jcbfm.9600546 17726492

[ref78] Li Y. Park J. S. Deng J. H. Bai Y. (2006). Cytochrome c oxidase subunit IV is essential for assembly and respiratory function of the enzyme complex. J. Bioenerg. Biomembr. 38, 283–291. doi: 10.1007/s10863-006-9052-z, PMID: 17091399 PMC1885940

[ref79] Li Z. Song Y. He T. Wen R. Li Y. Chen T.. (2021). M2 microglial small extracellular vesicles reduce glial scar formation via the miR-124/STAT3 pathway after ischemic stroke in mice. Theranostics 11, 1232–1248. doi: 10.7150/thno.48761, PMID: 33391532 PMC7738903

[ref80] Li L. Voloboueva L. Griffiths B. B. Xu L. Giffard R. G. Stary C. M. (2021). MicroRNA-338 inhibition protects against focal cerebral ischemia and preserves mitochondrial function in vitro in astrocytes and neurons via COX4I1. Mitochondrion 59, 105–112. doi: 10.1016/j.mito.2021.04.013, PMID: 33933660 PMC8292173

[ref81] Li D. Wang Y. Jin X. Hu D. Xia C. Xu H.. (2020). NK cell-derived exosomes carry miR-207 and alleviate depression-like symptoms in mice. J. Neuroinflammation 17:126. doi: 10.1186/s12974-020-01787-4, PMID: 32321532 PMC7178582

[ref82] Lian H. Litvinchuk A. Chiang A. C. Aithmitti N. Jankowsky J. L. Zheng H. (2016). Astrocyte-microglia cross talk through complement activation modulates amyloid pathology in mouse models of Alzheimer's disease. J. Neurosci. 36, 577–589. doi: 10.1523/JNEUROSCI.2117-15.2016, PMID: 26758846 PMC4710776

[ref83] Liddelow S. A. Barres B. A. (2017). Reactive astrocytes: production, function, and therapeutic potential. Immunity 46, 957–967. doi: 10.1016/j.immuni.2017.06.006 28636962

[ref84] Liddelow S. A. Guttenplan K. A. Clarke L. E. Bennett F. C. Bohlen C. J. Schirmer L.. (2017). Neurotoxic reactive astrocytes are induced by activated microglia. Nature 541, 481–487. doi: 10.1038/nature21029, PMID: 28099414 PMC5404890

[ref85] Liebner S. Dijkhuizen R. M. Reiss Y. Plate K. H. Agalliu D. Constantin G. (2018). Functional morphology of the blood-brain barrier in health and disease. Acta Neuropathol. 135, 311–336. doi: 10.1007/s00401-018-1815-1, PMID: 29411111 PMC6781630

[ref86] Lin S. Gregory R. I. (2015). MicroRNA biogenesis pathways in cancer. Nat. Rev. Cancer 15, 321–333. doi: 10.1038/nrc3932, PMID: 25998712 PMC4859809

[ref87] Litvinchuk A. Wan Y. W. Swartzlander D. B. Chen F. Cole A. Propson N. E.. (2018). Complement C3aR inactivation attenuates tau pathology and reverses an immune network deregulated in Tauopathy models and Alzheimer's disease. Neuron 100, 1337–1353.e5. doi: 10.1016/j.neuron.2018.10.031, PMID: 30415998 PMC6309202

[ref88] Liu L. R. Liu J. C. Bao J. S. Bai Q. Q. Wang G. Q. (2020). Interaction of microglia and astrocytes in the neurovascular unit. Front. Immunol. 11:1024. doi: 10.3389/fimmu.2020.01024, PMID: 32733433 PMC7362712

[ref89] Luna-Herrera C. Martínez-Dávila I. A. Soto-Rojas L. O. Flores-Martinez Y. M. Fernandez-Parrilla M. A. Ayala-Davila J.. (2020). Intranigral administration of β-Sitosterol-β-D-Glucoside elicits neurotoxic A1 astrocyte reactivity and chronic Neuroinflammation in the rat Substantia Nigra. J Immunol Res 2020:5907591. doi: 10.1155/2020/5907591, PMID: 33282962 PMC7685831

[ref90] Mac Vicar B. A. Newman E. A. (2015). Astrocyte regulation of blood flow in the brain. Cold Spring Harb. Perspect. Biol. 7:a020388. doi: 10.1101/cshperspect.a020388, PMID: 25818565 PMC4448617

[ref91] Madji Hounoum B. Mavel S. Coque E. Patin F. Vourc'h P. Marouillat S.. (2017). Wildtype motoneurons, ALS-linked SOD1 mutation and glutamate profoundly modify astrocyte metabolism and lactate shuttling. Glia 65, 592–605. doi: 10.1002/glia.23114, PMID: 28139855

[ref92] Magistretti P. J. Allaman I. (2018). Lactate in the brain: from metabolic end-product to signalling molecule. Nat. Rev. Neurosci. 19, 235–249. doi: 10.1038/nrn.2018.19, PMID: 29515192

[ref93] Makar T. K. Nedergaard M. Preuss A. Gelbard A. S. Perumal A. S. Cooper A. J. (1994). Vitamin E, ascorbate, glutathione, glutathione disulfide, and enzymes of glutathione metabolism in cultures of chick astrocytes and neurons: evidence that astrocytes play an important role in antioxidative processes in the brain. J. Neurochem. 62, 45–53. doi: 10.1046/j.1471-4159.1994.62010045.x, PMID: 7903354

[ref94] Manley G. T. Fujimura M. Ma T. Noshita N. Filiz F. Bollen A. W.. (2000). Aquaporin-4 deletion in mice reduces brain edema after acute water intoxication and ischemic stroke. Nat. Med. 6, 159–163. doi: 10.1038/72256, PMID: 10655103

[ref95] Marcos-Contreras O. A. Greineder C. F. Kiseleva R. Y. Parhiz H. Walsh L. R. Zuluaga-Ramirez V.. (2020). Selective targeting of nanomedicine to inflamed cerebral vasculature to enhance the blood-brain barrier. Proc. Natl. Acad. Sci. U. S. A. 117, 3405–3414. doi: 10.1073/pnas.1912012117, PMID: 32005712 PMC7035611

[ref96] Masamoto K. Unekawa M. Watanabe T. Toriumi H. Takuwa H. Kawaguchi H.. (2015). Unveiling astrocytic control of cerebral blood flow with optogenetics. Sci. Rep. 5:11455. doi: 10.1038/srep11455, PMID: 26076820 PMC4468581

[ref97] McKeon R. J. Schreiber R. C. Rudge J. S. Silver J. (1991). Reduction of neurite outgrowth in a model of glial scarring following CNS injury is correlated with the expression of inhibitory molecules on reactive astrocytes. J. Neurosci. 11, 3398–3411. doi: 10.1523/JNEUROSCI.11-11-03398.1991, PMID: 1719160 PMC6575543

[ref98] Meng X. Zhong J. Zeng C. Yung K. Zhang X. Wu X.. (2021). MiR-30a-5p regulates GLT-1 function via a PKCα-mediated ubiquitin degradation pathway in a mouse model of Parkinson's disease. ACS Chem. Neurosci. 12, 1578–1592. doi: 10.1021/acschemneuro.1c00076, PMID: 33882234

[ref99] Miklya I. Göltl P. Hafenscher F. Pencz N. (2014). The role of parkin in Parkinson's disease. Neuropsychopharmacol. Hung. 16, 67–76. 24978049

[ref100] Mizee M. R. Wooldrik D. Lakeman K. A. van het Hof B. Drexhage J. A. Geerts D.. (2013). Retinoic acid induces blood-brain barrier development. J. Neurosci. 33, 1660–1671. doi: 10.1523/JNEUROSCI.1338-12.2013, PMID: 23345238 PMC6618717

[ref101] Mo Y. Sun Y. Y. Liu K. Y. (2020). Autophagy and inflammation in ischemic stroke. Neural Regen. Res. 15, 1388–1396. doi: 10.4103/1673-5374.274331, PMID: 31997797 PMC7059569

[ref102] Montagne A. Nation D. A. Sagare A. P. Barisano G. Sweeney M. D. Chakhoyan A.. (2020). APOE4 leads to blood-brain barrier dysfunction predicting cognitive decline. Nature 581, 71–76. doi: 10.1038/s41586-020-2247-3, PMID: 32376954 PMC7250000

[ref103] Morrison B. M. Lee Y. Rothstein J. D. (2013). Oligodendroglia: metabolic supporters of axons. Trends Cell Biol. 23, 644–651. doi: 10.1016/j.tcb.2013.07.007, PMID: 23988427 PMC3842360

[ref104] Mukherjee N. Nandi S. Garg S. Ghosh S. Ghosh S. Samat R.. (2020). Targeting chondroitin sulfate proteoglycans: an emerging therapeutic strategy to treat CNS injury. ACS Chem. Neurosci. 11, 231–232. doi: 10.1021/acschemneuro.0c00004, PMID: 31939650

[ref105] Nakano M. Kubota K. Kobayashi E. Chikenji T. S. Saito Y. Konari N.. (2020). Bone marrow-derived mesenchymal stem cells improve cognitive impairment in an Alzheimer's disease model by increasing the expression of microRNA-146a in hippocampus. Sci. Rep. 10:10772. doi: 10.1038/s41598-020-67460-1, PMID: 32612165 PMC7330036

[ref106] Nesic O. Lee J. Ye Z. Unabia G. C. Rafati D. Hulsebosch C. E.. (2006). Acute and chronic changes in aquaporin 4 expression after spinal cord injury. Neuroscience 143, 779–792. doi: 10.1016/j.neuroscience.2006.08.079, PMID: 17074445 PMC1894918

[ref107] Noh J. H. Chang Y. G. Kim M. G. Jung K. H. Kim J. K. Bae H. J.. (2013). MiR-145 functions as a tumor suppressor by directly targeting histone deacetylase 2 in liver cancer. Cancer Lett. 335, 455–462. doi: 10.1016/j.canlet.2013.03.003, PMID: 23499894

[ref108] Norden D. M. Fenn A. M. Dugan A. Godbout J. P. (2014). TGFβ produced by IL-10 redirected astrocytes attenuates microglial activation. Glia 62, 881–895. doi: 10.1002/glia.22647, PMID: 24616125 PMC4061706

[ref109] Ohtake Y. Li S. (2015). Molecular mechanisms of scar-sourced axon growth inhibitors. Brain Res. 1619, 22–35. doi: 10.1016/j.brainres.2014.08.064, PMID: 25192646 PMC4345149

[ref110] Okada S. Nakamura M. Katoh H. Miyao T. Shimazaki T. Ishii K.. (2006). Conditional ablation of stat 3 or Socs 3 discloses a dual role for reactive astrocytes after spinal cord injury. Nat. Med. 12, 829–834. doi: 10.1038/nm1425 16783372

[ref111] Olanow C. W. Tatton W. G. (1999). Etiology and pathogenesis of Parkinson's disease. Annu. Rev. Neurosci. 22, 123–144. doi: 10.1146/annurev.neuro.22.1.123 10202534

[ref112] Ouyang Y. B. Voloboueva L. A. Xu L. J. Giffard R. G. (2007). Selective dysfunction of hippocampal CA1 astrocytes contributes to delayed neuronal damage after transient forebrain ischemia. J. Neurosci. 27, 4253–4260. doi: 10.1523/JNEUROSCI.0211-07.2007, PMID: 17442809 PMC3140959

[ref113] Peng G. Yuan Y. Wu S. He F. Hu Y. Luo B. (2015). MicroRNA let-7e is a potential circulating biomarker of acute stage ischemic stroke. Transl. Stroke Res. 6, 437–445. doi: 10.1007/s12975-015-0422-x, PMID: 26415639

[ref114] Peng L. Zhao Y. Li Y. Zhou Y. Li L. Lei S.. (2019). Effect of DJ-1 on the neuroprotection of astrocytes subjected to cerebral ischemia/reperfusion injury. J. Mol. Med. (Berl) 97, 189–199. doi: 10.1007/s00109-018-1719-5, PMID: 30506316 PMC6348070

[ref115] Pines G. Danbolt N. C. Bjørås M. Zhang Y. Bendahan A. Eide L.. (1992). Cloning and expression of a rat brain L-glutamate transporter. Nature 360, 464–467. doi: 10.1038/360464a0 1448170

[ref116] Plog B. A. Nedergaard M. (2018). The Glymphatic system in central nervous system health and disease: past, present, and future. Annu. Rev. Pathol. 13, 379–394. doi: 10.1146/annurev-pathol-051217-111018, PMID: 29195051 PMC5803388

[ref117] Polanco J. C. Li C. Bodea L. G. Martinez-Marmol R. Meunier F. A. Götz J. (2018). Amyloid-β and tau complexity - towards improved biomarkers and targeted therapies. Nat. Rev. Neurol. 14, 22–39. doi: 10.1038/nrneurol.2017.162, PMID: 29242522

[ref118] Querfurth H. W. LaFerla F. M. (2010). Alzheimer's disease. N. Engl. J. Med. 362, 329–344. doi: 10.1056/NEJMra0909142 20107219

[ref119] Ramandi D. Elahdadi Salmani M. Moghimi A. Lashkarbolouki T. Fereidoni M. (2021). Pharmacological upregulation of GLT-1 alleviates the cognitive impairments in the animal model of temporal lobe epilepsy. PLoS One 16:e0246068. doi: 10.1371/journal.pone.0246068, PMID: 33507976 PMC7842975

[ref120] Renault-Mihara F. Mukaino M. Shinozaki M. Kumamaru H. Kawase S. Baudoux M.. (2017). Regulation of rho a by STAT3 coordinates glial scar formation. J. Cell Biol. 216, 2533–2550. doi: 10.1083/jcb.201610102, PMID: 28642362 PMC5551705

[ref121] Rolls A. Shechter R. Schwartz M. (2009). The bright side of the glial scar in CNS repair. Nat. Rev. Neurosci. 10, 235–241. doi: 10.1038/nrn2591, PMID: 19229242

[ref122] Rupaimoole R. Calin G. A. Lopez-Berestein G. Sood A. K. (2016). miRNA deregulation in cancer cells and the tumor microenvironment. Cancer Discov. 6, 235–246. doi: 10.1158/2159-8290.CD-15-0893, PMID: 26865249 PMC4783232

[ref123] Schousboe A. Bak L. K. Waagepetersen H. S. (2013). Astrocytic control of biosynthesis and turnover of the neurotransmitters glutamate and GABA. Front Endocrinol (Lausanne) 4:102. doi: 10.3389/fendo.2013.00102, PMID: 23966981 PMC3744088

[ref124] Sekar A. Bialas A. R. de Rivera H. Davis A. Hammond T. R. Kamitaki N.. (2016). Schizophrenia risk from complex variation of complement component 4. Nature 530, 177–183. doi: 10.1038/nature16549, PMID: 26814963 PMC4752392

[ref125] Shen Y. Tenney A. P. Busch S. A. Horn K. P. Cuascut F. X. Liu K.. (2009). PTPsigma is a receptor for chondroitin sulfate proteoglycan, an inhibitor of neural regeneration. Science 326, 592–596. doi: 10.1126/science.1178310, PMID: 19833921 PMC2811318

[ref126] Shi Q. Chowdhury S. Ma R. Le K. X. Hong S. Caldarone B. J.. (2017). Complement C3 deficiency protects against neurodegeneration in aged plaque-rich APP/PS1 mice. Sci. Transl. Med. 9:eaaf6295. doi: 10.1126/scitranslmed.aaf6295, PMID: 28566429 PMC6936623

[ref127] Shiow L. R. Favrais G. Schirmer L. Schang A. L. Cipriani S. Andres C.. (2017). Reactive astrocyte COX2-PGE2 production inhibits oligodendrocyte maturation in neonatal white matter injury. Glia 65, 2024–2037. doi: 10.1002/glia.23212, PMID: 28856805 PMC5753598

[ref128] Silver J. Miller J. H. (2004). Regeneration beyond the glial scar. Nat. Rev. Neurosci. 5, 146–156. doi: 10.1038/nrn1326 14735117

[ref129] Skowrońska K. Obara-Michlewska M. Zielińska M. Albrecht J. (2019). NMDA receptors in astrocytes: in search for roles in neurotransmission and Astrocytic homeostasis. Int. J. Mol. Sci. 20:309. doi: 10.3390/ijms20020309, PMID: 30646531 PMC6358855

[ref130] Sofroniew M. V. (2009). Molecular dissection of reactive astrogliosis and glial scar formation. Trends Neurosci. 32, 638–647. doi: 10.1016/j.tins.2009.08.002, PMID: 19782411 PMC2787735

[ref131] Sofroniew M. V. Vinters H. V. (2010). Astrocytes: biology and pathology. Acta Neuropathol. 119, 7–35. doi: 10.1007/s00401-009-0619-8, PMID: 20012068 PMC2799634

[ref132] Song S. Huang H. Guan X. Fiesler V. Bhuiyan M. Liu R.. (2021). Activation of endothelial Wnt/β-catenin signaling by protective astrocytes repairs BBB damage in ischemic stroke. Prog. Neurobiol. 199:101963. doi: 10.1016/j.pneurobio.2020.101963, PMID: 33249091 PMC7925353

[ref133] Srivastava I. Vazquez-Juarez E. Henning L. Gómez-Galán M. Lindskog M. (2020). Blocking Astrocytic GABA restores synaptic plasticity in prefrontal cortex of rat model of depression. Cells 9:1705. doi: 10.3390/cells9071705, PMID: 32708718 PMC7408154

[ref134] Stary C. M. Sun X. Ouyang Y. Li L. Giffard R. G. (2016). miR-29a differentially regulates cell survival in astrocytes from cornu ammonis 1 and dentate gyrus by targeting VDAC1. Mitochondrion 30, 248–254. doi: 10.1016/j.mito.2016.08.013, PMID: 27553862 PMC6082172

[ref135] Stephan A. H. Barres B. A. Stevens B. (2012). The complement system: an unexpected role in synaptic pruning during development and disease. Annu. Rev. Neurosci. 35, 369–389. doi: 10.1146/annurev-neuro-061010-113810, PMID: 22715882

[ref136] Storck T. Schulte S. Hofmann K. Stoffel W. (1992). Structure, expression, and functional analysis of a Na(+)-dependent glutamate/aspartate transporter from rat brain. Proc. Natl. Acad. Sci. U. S. A. 89, 10955–10959. doi: 10.1073/pnas.89.22.10955, PMID: 1279699 PMC50461

[ref137] Tanaka K. Watase K. Manabe T. Yamada K. Watanabe M. Takahashi K.. (1997). Epilepsy and exacerbation of brain injury in mice lacking the glutamate transporter GLT-1. Science 276, 1699–1702. doi: 10.1126/science.276.5319.1699, PMID: 9180080

[ref138] Ter Horst R. Jaeger M. Smeekens S. P. Oosting M. Swertz M. A. Li Y.. (2016). Host and environmental factors influencing individual human cytokine responses. Cells 167, 1111–1124.e13. doi: 10.1016/j.cell.2016.10.018, PMID: 27814508 PMC5787854

[ref139] Thrane A. S. Rappold P. M. Fujita T. Torres A. Bekar L. K. Takano T.. (2011). Critical role of aquaporin-4 (AQP4) in astrocytic Ca2+ signaling events elicited by cerebral edema. Proc. Natl. Acad. Sci. U. S. A. 108, 846–851. doi: 10.1073/pnas.1015217108, PMID: 21187412 PMC3021020

[ref140] Tsai H. H. Li H. Fuentealba L. C. Molofsky A. V. Taveira-Marques R. Zhuang H.. (2012). Regional astrocyte allocation regulates CNS synaptogenesis and repair. Science 337, 358–362. doi: 10.1126/science.1222381, PMID: 22745251 PMC4059181

[ref141] Tu Z. Li Y. Dai Y. Li L. Lv G. Chen I.. (2017). MiR-140/BDNF axis regulates normal human astrocyte proliferation and LPS-induced IL-6 and TNF-α secretion. Biomed. Pharmacother. 91, 899–905. doi: 10.1016/j.biopha.2017.05.016, PMID: 28501777

[ref142] Van Damme P. Bogaert E. Dewil M. Hersmus N. Kiraly D. Scheveneels W.. (2007). Astrocytes regulate Glu R2 expression in motor neurons and their vulnerability to excitotoxicity. Proc. Natl. Acad. Sci. U. S. A. 104, 14825–14830. doi: 10.1073/pnas.0705046104, PMID: 17804792 PMC1976195

[ref143] Verdejo H. E. del Campo A. Troncoso R. Gutierrez T. Toro B. Quiroga C.. (2012). Mitochondria, myocardial remodeling, and cardiovascular disease. Curr. Hypertens. Rep. 14, 532–539. doi: 10.1007/s11906-012-0305-4 22972531

[ref144] Verkhratsky A. Nedergaard M. (2018). Physiology of Astroglia. Physiol. Rev. 98, 239–389. doi: 10.1152/physrev.00042.2016, PMID: 29351512 PMC6050349

[ref145] Verkman A. S. Hara-Chikuma M. Papadopoulos M. C. (2008). Aquaporins--new players in cancer biology. J. Mol. Med. (Berl) 86, 523–529. doi: 10.1007/s00109-008-0303-9, PMID: 18311471 PMC3590015

[ref146] Wang Y. Cheng X. He Q. Zheng Y. Kim D. H. Whittemore S. R.. (2011). Astrocytes from the contused spinal cord inhibit oligodendrocyte differentiation of adult oligodendrocyte precursor cells by increasing the expression of bone morphogenetic proteins. J. Neurosci. 31, 6053–6058. doi: 10.1523/JNEUROSCI.5524-09.2011, PMID: 21508230 PMC3081104

[ref147] Wang L. Wu L. Duan Y. Xu S. Yang Y. Yin J.. (2022). Phenotype shifting in astrocytes account for benefits of intra-arterial selective cooling infusion in hypertensive rats of ischemic stroke. Neurotherapeutics 19, 386–398. doi: 10.1007/s13311-022-01186-y, PMID: 35044645 PMC9130426

[ref148] Wang C. Y. Yang S. H. Tzeng S. F. (2015). MicroRNA-145 as one negative regulator of astrogliosis. Glia 63, 194–205. doi: 10.1002/glia.22743, PMID: 25139829

[ref149] Wang H. Zheng X. Jin J. Zheng L. Guan T. Huo Y.. (2020). LncRNA MALAT1 silencing protects against cerebral ischemia-reperfusion injury through miR-145 to regulate AQP4. J. Biomed. Sci. 27:40. doi: 10.1186/s12929-020-00635-0, PMID: 32138732 PMC7059719

[ref150] Wanner I. B. Anderson M. A. Song B. Levine J. Fernandez A. Gray-Thompson Z.. (2013). Glial scar borders are formed by newly proliferated, elongated astrocytes that interact to corral inflammatory and fibrotic cells via STAT3-dependent mechanisms after spinal cord injury. J. Neurosci. 33, 12870–12886. doi: 10.1523/JNEUROSCI.2121-13.2013, PMID: 23904622 PMC3728693

[ref151] Weiss N. Miller F. Cazaubon S. Couraud P. O. (2009). The blood-brain barrier in brain homeostasis and neurological diseases. Biochim. Biophys. Acta 1788, 842–857. doi: 10.1016/j.bbamem.2008.10.022 19061857

[ref152] Weller M. L. Stone I. M. Goss A. Rau T. Rova C. Poulsen D. J. (2008). Selective overexpression of excitatory amino acid transporter 2 (EAAT2) in astrocytes enhances neuroprotection from moderate but not severe hypoxia-ischemia. Neuroscience 155, 1204–1211. doi: 10.1016/j.neuroscience.2008.05.059, PMID: 18620031 PMC2729515

[ref153] Wolters F. J. Ikram M. A. (2018). Epidemiology of dementia: the burden on society, the challenges for research. Methods Mol. Biol. 1750, 3–14. doi: 10.1007/978-1-4939-7704-8_1 29512062

[ref154] Worringer K. A. Rand T. A. Hayashi Y. Sami S. Takahashi K. Tanabe K.. (2014). The let-7/LIN-41 pathway regulates reprogramming to human induced pluripotent stem cells by controlling expression of prodifferentiation genes. Cell Stem Cell 14, 40–52. doi: 10.1016/j.stem.2013.11.001, PMID: 24239284 PMC3982312

[ref155] Wosik K. Cayrol R. Dodelet-Devillers A. Berthelet F. Bernard M. Moumdjian R.. (2007). Angiotensin II controls occludin function and is required for blood brain barrier maintenance: relevance to multiple sclerosis. J. Neurosci. 27, 9032–9042. doi: 10.1523/JNEUROSCI.2088-07.2007, PMID: 17715340 PMC6672193

[ref156] Wu X. Meng X. Tan F. Jiao Z. Zhang X. Tong H.. (2019). Regulatory mechanism of miR-543-3p on GLT-1 in a mouse model of Parkinson's disease. ACS Chem. Neurosci. 10, 1791–1800. doi: 10.1021/acschemneuro.8b00683, PMID: 30676715

[ref157] Xiang J. Tang Y. Li C. Su E. J. Lawrence D. A. Keep R. F. (2016). Mechanisms underlying astrocyte Endfeet swelling in stroke. Acta Neurochir. Suppl. 121, 19–22. doi: 10.1007/978-3-319-18497-5_4, PMID: 26463917

[ref158] Xu P. Sriramula S. Lazartigues E. (2011). ACE2/ANG-(1-7)/mas pathway in the brain: the axis of good. Am. J. Physiol. Regul. Integr. Comp. Physiol. 300, R804–R817. doi: 10.1152/ajpregu.00222.2010, PMID: 21178125 PMC3075080

[ref159] Yamada T. Kawahara K. Kosugi T. Tanaka M. (2006). Nitric oxide produced during sublethal ischemia is crucial for the preconditioning-induced down-regulation of glutamate transporter GLT-1 in neuron/astrocyte co-cultures. Neurochem. Res. 31, 49–56. doi: 10.1007/s11064-005-9077-4, PMID: 16474996

[ref160] Yang Z. B. Luo X. J. Ren K. D. Peng J. J. Tan B. Liu B.. (2015). Beneficial effect of magnesium lithospermate B on cerebral ischemia-reperfusion injury in rats involves the regulation of miR-107/glutamate transporter 1 pathway. Eur. J. Pharmacol. 766, 91–98. doi: 10.1016/j.ejphar.2015.09.042, PMID: 26420356

[ref161] Yang Z. B. Zhang Z. Li T. B. Lou Z. Li S. Y. Yang H.. (2014). Up-regulation of brain-enriched miR-107 promotes excitatory neurotoxicity through down-regulation of glutamate transporter-1 expression following ischaemic stroke. Clin. Sci. (Lond) 127, 679–689. doi: 10.1042/CS20140084, PMID: 24943094

[ref162] Ye Y. Hao J. Hong Z. Wu T. Ge X. Qian B.. (2022). Downregulation of MicroRNA-145-5p in activated microglial Exosomes promotes astrocyte proliferation by removal of Smad 3 inhibition. Neurochem. Res. 47, 382–393. doi: 10.1007/s11064-021-03446-3, PMID: 34623564

[ref163] Yeh T. H. Lee D. Y. Gianino S. M. Gutmann D. H. (2009). Microarray analyses reveal regional astrocyte heterogeneity with implications for neurofibromatosis type 1 (NF1)-regulated glial proliferation. Glia 57, 1239–1249. doi: 10.1002/glia.20845, PMID: 19191334 PMC2706934

[ref164] Zamanian J. L. Xu L. Foo L. C. Nouri N. Zhou L. Giffard R. G.. (2012). Genomic analysis of reactive astrogliosis. J. Neurosci. 32, 6391–6410. doi: 10.1523/JNEUROSCI.6221-11.2012, PMID: 22553043 PMC3480225

[ref165] Zeevalk G. D. Davis N. Hyndman A. G. Nicklas W. J. (1998). Origins of the extracellular glutamate released during total metabolic blockade in the immature retina. J. Neurochem. 71, 2373–2381. doi: 10.1046/j.1471-4159.1998.71062373.x, PMID: 9832135

[ref166] Zeisel A. Muñoz-Manchado A. B. Codeluppi S. Lönnerberg P. La Manno G. Juréus A.. (2015). Brain structure. Cell types in the mouse cortex and hippocampus revealed by single-cell RNA-seq. Science 347, 1138–1142. doi: 10.1126/science.aaa1934, PMID: 25700174

[ref167] Zeng X. Dong X. Xiao Q. Yao J. (2022). Vitamin C inhibits Ubiquitination of glutamate transporter 1 (GLT-1) in astrocytes by Downregulating HECTD1. ACS Chem. Neurosci. 13, 676–687. doi: 10.1021/acschemneuro.1c00845, PMID: 35148069

[ref168] Zeng H. K. Wang Q. S. Deng Y. Y. Fang M. Chen C. B. Fu Y. H.. (2010). Hypertonic saline ameliorates cerebral edema through downregulation of aquaporin-4 expression in the astrocytes. Neuroscience 166, 878–885. doi: 10.1016/j.neuroscience.2009.12.076, PMID: 20083168

[ref169] Zhang Y. Barres B. A. (2010). Astrocyte heterogeneity: an underappreciated topic in neurobiology. Curr. Opin. Neurobiol. 20, 588–594. doi: 10.1016/j.conb.2010.06.005, PMID: 20655735

[ref170] Zhang C. Chen J. Lu H. (2015). Expression of aquaporin-4 and pathological characteristics of brain injury in a rat model of traumatic brain injury. Mol. Med. Rep. 12, 7351–7357. doi: 10.3892/mmr.2015.4372, PMID: 26459070 PMC4626127

[ref171] Zhang Y. He X. Wu X. Lei M. Wei Z. Zhang X.. (2017). Rapamycin upregulates glutamate transporter and IL-6 expression in astrocytes in a mouse model of Parkinson's disease. Cell Death Dis. 8:e2611. doi: 10.1038/cddis.2016.491, PMID: 28182002 PMC5386462

[ref172] Zhang Y. Meng T. Chen J. Zhang Y. Kang J. Li X.. (2021). miR-21a-5p promotes inflammation following traumatic spinal cord injury through Upregulation of neurotoxic reactive astrocyte (A1) polarization by inhibiting the CNTF/STAT3/Nkrf pathway. Int. J. Biol. Sci. 17, 2795–2810. doi: 10.7150/ijbs.60509, PMID: 34345208 PMC8326122

[ref173] Zhang L. Y. Pan J. Mamtilahun M. Zhu Y. Wang L. Venkatesh A.. (2020). Microglia exacerbate white matter injury via complement C3/C3aR pathway after hypoperfusion. Theranostics 10, 74–90. doi: 10.7150/thno.35841, PMID: 31903107 PMC6929610

[ref174] Zhang Y. Xu D. Qi H. Yuan Y. Liu H. Yao S.. (2018). Enriched environment promotes post-stroke neurogenesis through NF-κB-mediated secretion of IL-17A from astrocytes. Brain Res. 1687, 20–31. doi: 10.1016/j.brainres.2018.02.030, PMID: 29481794

[ref175] Zhao F. Qu Y. Wang H. Huang L. Zhu J. Li S.. (2017). The effect of miR-30d on apoptosis and autophagy in cultured astrocytes under oxygen-glucose deprivation. Brain Res. 1671, 67–76. doi: 10.1016/j.brainres.2017.06.011, PMID: 28647273

[ref176] Zheng J. Lu J. Mei S. Wu H. Sun Z. Fang Y.. (2021). Ceria nanoparticles ameliorate white matter injury after intracerebral hemorrhage: microglia-astrocyte involvement in remyelination. J. Neuroinflammation 18:43. doi: 10.1186/s12974-021-02101-6, PMID: 33588866 PMC7883579

[ref177] Zheng Y. Wang L. Chen M. Pei A. Xie L. Zhu S. (2017). Upregulation of miR-130b protects against cerebral ischemic injury by targeting water channel protein aquaporin 4 (AQP4). Am. J. Transl. Res. 10, 5209–5224. doi: 10.7150/thno.43640, PMID: 28804561 PMC5527259

[ref178] Zhou Y. Danbolt N. C. (2013). GABA and glutamate transporters in brain. Front Endocrinol (Lausanne) 4:165. doi: 10.3389/fendo.2013.00165, PMID: 24273530 PMC3822327

[ref179] Zhou D. Huang Z. Zhu X. Hong T. Zhao Y. (2021). Circular RNA 0025984 ameliorates ischemic stroke injury and protects astrocytes through miR-143-3p/TET1/ORP150 pathway. Mol. Neurobiol. 58, 5937–5953. doi: 10.1007/s12035-021-02486-8, PMID: 34435328

[ref180] Zou L. H. Shi Y. J. He H. Jiang S. M. Huo F. F. Wang X. M.. (2019). Effects of FGF2/FGFR1 pathway on expression of A1 astrocytes after infrasound exposure. Front. Neurosci. 13:429. doi: 10.3389/fnins.2019.00429, PMID: 31130839 PMC6509904

